# Abstracts and short papers from the 5th Congress of the Polish Thyroid Association, Poznan, 3-5 September, 2015: Poznan, Poland. 3-5 September 2015

**DOI:** 10.1186/s13044-016-0035-9

**Published:** 2016-12-06

**Authors:** Leonard Wartofsky, Peter Smyth, Josef Köhrle, Ryszard Anielski, Marian Slowiaczek, Pawel Orlicki, Rafal Czepczynski, Jacek Daroszewski, Alicja Hubalewska-Dydejczyk, Aldona Kowalska, Maria Kurowska, Joanna Malicka, Anna Oszywa-Chabros, Agnieszka Zwolak, Jerzy S. Tarach, Krzysztof C. Lewandowski, Andrzej Lewinski, Anna Szeliga, Adam Czyzyk, Przemyslaw Niedzielski, Miroslaw Mleczek, Adam Maciejewski, Anna Oczkowska, Jolanta Dorszewska, Katarzyna Lacka, Jerzy S. Tarach, Elzbieta Andrysiak-Mamos, Elzbieta Sowinska-Przepiera, Ewa Zochowska, Bartosz Kiedrowicz, Agnieszka Kazmierczyk-Puchalska, Anhelli Syrenicz, Katarzyna D. Arczewska, Joanna Drozdowska, Wanda Krasuska, Anna Stachurska, Grazyna Hoser, Miroslaw Kiedrowski, Tomasz Stepien, Hilde Nilsen, Barbara Czarnocka, Karolina H. Czarnecka, Michal Kusinski, Agnieszka Nadel, Justyna Kiszalkiewicz, Daria Domanska, Monika Migdalska-Sek, Dorota Pastuszak-Lewandoska, Ewa Nawrot, Krzysztof Kuzdak, Ewa Brzezianska-Lasota, Barbara Czarnocka, Anna Maria Dabrowska, Jolanta Kijek, Jerzy S. Tarach, Anna Torun-Jurkowska, Beata Chrapko, Anna Maria Dabrowska, Jerzy S. Tarach, Jolanta Kijek, Helena Jastrzebska, Magdalena Kochman, Ewa Szczepanska, Joanna Zgliczynska-Widlak, Agnieszka Samsel, Wojciech Zgliczynski, Roman Junik, Dariusz Kajdaniuk, Grzegorz Kaminski, Grzegorz Kaminski, Krzysztof Giejda, Malgorzata Karbownik-Lewinska, Magdalena Marcinkowska, Jan Stepniak, Andrzej Lewinski, Monika Koziolek, Anna Sieradzka, Magdalena Lewandowska, Maria Stepaniuk, Bartosz Kiedrowicz, Julita Duda, Elzbieta Andrysiak-Mamos, Anhelli Syrenicz, Monika Koziolek, Anna Sieradzka, Ewa Wentland-Kotwicka, Bartosz Kiedrowicz, Milosz Parczewski, Maria Stepaniuk, Agnieszka Binczak-Kuleta, Andrzej Ciechanowicz, Elzbieta Andrysiak-Mamos, Anhelli Syrenicz, Maria Kurowska, Joanna Malicka, Piotr Denew, Agnieszka Zwolak, Monika Lenart-Lipinska, Jerzy S. Tarach, Katarzyna Lacka, Dagny Lapinska, Kosma Wolinski, Magdalena Matysiak-Grzes, Aleksandra Klimowicz, Edyta Gurgul, Rafal Czepczynski, Maria Gryczynska, Marek Ruchala, Hanna Mikos, Marcin Mikos, Barbara Rabska-Pietrzak, Marek Niedziela, Marek Ruchala, Ewelina Szczepanek-Parulska, Magdalena Rudzinska, Joanna Ledwon, Kamila Karpinska, Maria Macios, Justyna Sikorska, Barbara Czarnocka, Malgorzata Ruminska, Ewelina Witkowska-Sedek, Beata Pyrzak, Nadia Sawicka-Gutaj, Anna Sieradzka, Monika Koziołek, Magdalena Lewandowska, Ewa Wentland-Kotwicka, Marcin Machaj, Lilianna Osowicz-Korolonek, Jakub Pobłocki, Anhelli Syrenicz, Jerzy Sowinski, Nadia Sawicka-Gutaj, Paulina Ziolkowska, Marek Ruchala, Ewelina Szczepanek-Parulska, Bartlomiej Budny, Marek Ruchala, Malgorzata Trofimiuk-Muldner, Katarzyna Ziemnicka, Dorota Zozulinska-Ziolkiewicz

**Affiliations:** 1 Georgetown University School of Medicine, MedStar Washington Hospital Center, Washington, D.C. USA; 2 University College Dublin, Dublin, Republic of Ireland; 3 National University of Ireland, Galway, Republic of Ireland; 4 Institut für Experimentelle Endokrinologie, Charité-Universitätsmedizin Berlin, Berlin, Germany; 5 Department of Short-stay Surgery, St. John Grande Hospital of Brothers Hospitallers, Cracow, Poland; 6 Department of Catastrophe Medicine and Emergency Medicine, Chair of Anesthesiology and Intensive Care, Jagiellonian University, Medical College, Cracow, Poland; 7 Department of Endocrinology, Metabolism and Internal Diseases, Poznan University of Medical Sciences, Poznan, Poland; 8 Department of Endocrinology, Diabetes and Isotope Therapy, Medical University, Wroclaw, Poland; 9 Chair and Department of Endocrinology, Jagiellonian University, Medical College, Cracow, Poland; 10 Endocrinology Department, Holycross Cancer Centre, Kielce, Poland; 11 Department of Endocrinology, Medical University of Lublin, Lublin, Poland; 12 Department of Endocrinology and Metabolic Diseases, The Medical University of Lodz, Lodz, Poland; 13 Department of Endocrinology and Metabolic Diseases, Medical University of Lodz, Lodz, Poland; 14 Student Scientific Society, Poznan University of Medical Sciences, Poznan, Poland; 15 Department of Gynecological Endocrinology, Poznan University of Medical Sciences, Poznan, Poland; 16 Department of Analytical Chemistry, Adam Mickiewicz University in Poznan, Poznan, Poland; 17 Department of Chemistry, Poznan University of Life Sciences, Poznan, Poland; 18 Department of Endocrinology, Metabolism and Internal Medicine, Poznan University of Medical Sciences, Poznan, Poland; 19 Laboratory of Neurobiology, Department of Neurology, Poznan University of Medical Sciences, Poznan, Poland; 20 Department of Endocrinology, Medical University, Lublin, Poland; 21 Department of Endocrinology, Metabolic and Internal Diseases, Pomeranian Medical University, Szczecin, Poland; 22 Department of Biochemistry and Molecular Biology, The Centre of Postgraduate Medical Education, Warsaw, Poland; 23 Department of Immunohematology, The Centre of Postgraduate Medical Education, Warsaw, Poland; 24 Department of Clinical Cytology, The Centre of Postgraduate Medical Education, Warsaw, Poland; 25 Department of Pathology, Maria Sklodowska-Curie Memorial Cancer Centre and Institute of Oncology, Warsaw, Poland; 26 Department of General and Endocrinological Surgery, Copernicus Voivodship Hospital, Lodz, Poland; 27 Department of Clinical Molecular Biology, University of Oslo, Oslo, Norway; 28 Department of Molecular Bases of Medicine, I Chair of Internal Medicine, Medical University of Lodz, Lodz, Poland; 29 Department of Endocrine, General and Vascular Surgery, Chair of Endocrinology, Medical University of Lodz, Lodz, Poland; 30 Department of Biochemistry and Molecular Biology, The Centre of Postgraduate Medical Education, Warsaw, Poland; 31 Department of Endocrinology, Medical University of Lublin, Lublin, Poland; 32 Department of Nuclear Medicine, Medical University of Lublin, Lublin, Poland; 33 Department of Mathematics and Medical Biostatistics, Medical University of Lublin, Lublin, Poland; 34 Department of Endocrinology, Medical University of Lublin, Lublin, Poland; 35 Department of Nuclear Medicine, Medical University of Lublin, Lublin, Poland; 36 Endocrinology Department of Medical Centre of Postgraduate Education, Warsaw, Poland; 37 Ophthalmology Department, Childrens Hospital, Warsaw, Poland; 38 Division of Endocrinology and Diabetology, L. Rydygier Collegium Medicum in Bydgoszcz, Bydgoszcz, Poland, Nicolaus Copernicus University in Torun, Torun, Poland; 39 Department of Pathophysiology and Endocrinology, Medical University of Silesia in Katowice, Katowice, Poland; 40 Department of Endocrinology and Diabetology, Voivodship Specialistic Hospital No. 3 in Rybnik, Rybnik, Poland; 41 Department of Nuclear Medicine and Endocrine Oncology, Center of Oncology - Maria Sklodowska-Curie Memorial Institute, Branch in Gliwice, Gliwice, Poland; 42 Department of Endocrinology and Isotope Therapy, Military Institute of Medicine, Warsaw, Poland; 43 Department of Endocrinology and Isotope Therapy, Military Institute of Medicine, Warsaw, Poland; 44 Polish Mother’s Memorial Hospital – Research Institute, Lodz, Poland; 45 Department of Oncological Endocrinology, Medical University of Lodz, Lodz, Poland; 46 Department of Endocrinology and Metabolic Diseases, Medical University of Lodz, Lodz, Poland; 47 Department of Endocrinology, Metabolic and Internal Diseases, Pomeranian Medical University, Szczecin, Poland; 48 Department of Pathology, Pomeranian Medical University, Szczecin, Poland; 49 West Pomeranian Oncology Center, Szczecin, Poland; 50 Pomeranian Medical University Clinical Hospital No. 1, Szczecin, Poland; 51 Department of Endocrinology, Metabolic and Internal Diseases, Pomeranian Medical University in Szczecin, Szczecin, Poland; 52 Department of Infectious, Tropical Diseases and Immune Deficiency, Pomeranian Medical University in Szczecin, Szczecin, Poland; 53 Department of Pathology, Pomeranian Medical University in Szczecin, Szczecin, Poland; 54 West Pomeranian Oncology Center, Szczecin, Poland; 55 Department of Clinical and Molecular Biochemistry, Pomeranian Medical University in Szczecin, Szczecin, Poland; 56 Department of Endocrinology, Medical University of Lublin, Lublin, Poland; 57 Department of Endocrinology, Metabolism and Internal Medicine, University of Medical Sciences, Poznan, Poland; 58 Department of Endocrinology, Metabolism and Internal Medicine, Poznan University of Medical Sciences, Poznan, Poland; 59 Molecular Endocrinology Laboratory, Department of Pediatric Endocrinology and Rheumatology, 2nd Chair of Pediatrics, Poznan University of Medical Sciences, Poznan, Poland; 60 Department of Pediatric Endocrinology and Rheumatology, 2nd Chair of Pediatrics, Poznan University of Medical Sciences, Poznan, Poland; 61 Department of Pneumology, Allergology and Clinical Immunology, 3nd Chair of Pediatrics, Poznan University of Medical Sciences, Poznan, Poland; 62 Department of Endocrinology, Metabolism and Internal Medicine, Poznan University of Medical Sciences, Poznan, Poland; 63 Department of Biochemistry and Molecular Biology, Center of Postgraduate Medical Education, Warsaw, Poland; 64 Department of Pediatrics and Endocrinology, Medical University of Warsaw, Warsaw, Poland; 65 Department of Endocrinology, Metabolism and Internal Medicine, Poznan University of Medical Sciences, Poznan, Poland; 66 Department of Endocrinology, Metabolic and Internal Diseases, Pomeranian Medical University, Szczecin, Poland; 67 Department of Pathology, Pomeranian Medical University, Szczecin, Poland; 68 Department of Endocrinology and Internal Diseases, Autonomous Public Regional Joint Hospital in Szczecin, Szczecin, Poland; 69 Department of Endocrinology, Metabolism and Internal Medicine, Poznan University of Medical Sciences, Poznan, Poland; 70 Department of Endocrinology, Metabolism and Internal Medicine, Poznan University of Medical Sciences, Poznan, Poland; 71 Department of Endocrinology, Jagiellonian University Medical College, Cracow, Poland; 72 Endocrinology Department, University Hospital in Cracow, Cracow, Poland; 73 Department of Endocrinology, Metabolism and Internal Diseases, Poznan University of Medical Sciences, Poznan, Poland; 74 Department of Internal Medicine and Diabetology, Poznan University of Medical Sciences, Poznan, Poland

## Invited lectures: short papers

### A1 2016 ATA guidelines, risk assessment and management of thyroid cancer

#### Leonard Wartofsky

##### Georgetown University School of Medicine, MedStar Washington Hospital Center, Washington, D.C., USA

A Committee of the American Thyroid Association (ATA) recently completed an up to date revision of Guidelines for the Management of Thyroid Nodules and Differentiated Thyroid Cancer [1]. The publication is voluminous with some 100 different recommendations, each of which is supported by summaries of the evidence available in the literature. This lecture could describe a few highlights of the many important recommendations. In each case, suggested management was indicated by both the strength of the recommendation (strong, weak, or none) and the quality of the evidence (low, moderate, high, or insufficient). For example, in a patient with a nodule about to undergo surgery, it is recommended that a pre-operative ultrasound of the neck be performed to delineate the contralateral lobe and the lymph nodes in the central and lateral neck compartments, and this was a ‘strong’ recommendation based on ‘moderate’ quality evidence. A significant departure from earlier guidelines is the recommendation that lobectomy rather than total thyroidectomy can suffice for nodules that are indeterminate on FNA (AUS/FLUS, follicular neoplasm) and also for nodules of <1 cm even with biopsy proven cancer. However, total thyroidectomy is still recommended for indeterminate nodules >4 cm, FNA positive thyroid cancers >1 cm, nodules for which the FNA shows marked atypia, or nodules in patients with a family history of thyroid cancer or a history of radiation exposure to the neck. A summarized and practical approach to management of nodules appears in our recent report [2].

In addition to the use of AJCC/UICC staging of tumours, the Guidelines recommend that risk of recurrence be predicted with the 3-tiered ATA system of “low risk”, “intermediate risk”, and “high risk”. Another area of departure from earlier guidelines is the recommendation that 131-Iodine ablation is not necessary for most low risk tumours and even some less aggressive intermediate risk tumours, but clearly is recommended for high risk tumours. A decision for or against ablation may depend upon the post-operative status as indicated by the suppressed or stimulated serum level of thyroglobulin. The utility of post-operative, pre-ablation scans is discussed, with 123-Iodine preferable to 131-Iodine in order to avoid stunning. In regard to 131-Iodine therapy, lower doses are now recommended on the basis of studies by Mallick et al. [3] and Schlumberger et al. [4] indicating that 30 mCi (1.11 GBq) is as effective for ablation as are higher dose activities. Moreover, the recommendation is that ablation should be done when feasible after facilitation by recombinant human TSH, at least when given for low and intermediate risk patients.

Finally, the Guidelines focus on the importance of dynamic risk assessment or essentially a re-staging during the long term follow-up of our patients. Patients are categorized by this method as either having had an “excellent” response, a “biochemically incomplete” response, a “structurally incomplete” response, or an “indeterminate” response. Based upon this ongoing re-assessment, physicians will follow appropriately matched measures for either further diagnostic procedures or for treatment.

**Declarations**

**Ethics approval and consent to participate**

Not applicable

**Consent for publication**

Not applicable

**Availability of data and materials**

Not applicable

**Competing interests**

The author declares no conflict of interests.

**Funding**

Not applicable

**Peer review**

This short paper underwent the journal’s standard peer review process for supplements.

**References**

1. Haugen B Haugen BR, Alexander EK, Bible KC, Doherty GM, Mandel SJ, Nikiforov Y, Pacini F, Randolph G, Sawka A, Shepard D, Sosa J, Tuttle RM, Wartofsky L, 2015 American Thyroid Association Management Guidelines for adult patients with thyroid nodules and differentiated thyroid cancer. Available online at Thyroid 25: doi:10.1089/thy.2015.0020; print version in Thyroid 26:2016.

2. Burman KD, Wartofsky L. The thyroid nodule. New Engl J Med. 2015;373:2347-2356.

3. Mallick U, Harmer C, Yap B, Wadsley J, Clarke S, Moss L, Nicol A, Clark PM, Farnell K, McCready R, Smellie J, Franklyn JA, John R, Nutting CM, Newbold K, Lemon C, Gerrard G, Abdel-Hamid A, Hardman J, Macias E, Roques T, Whitaker S, Vijayan R, Alvarez P, Beare S, Forsyth S, Kadalayil L, Hackshaw A. Ablation with low-dose radioiodine and thyrotropin alfa in thyroid cancer. N Engl J Med. 2012;366:1674-1685.

4. Schlumberger M, Catargi B, Borget I, Deandreis D, Zerdoud S, Bridji B, Bardet S, Leenhardt L, Bastie D, Schvartz C, Vera P, Morel O, Benisvy D, Bournaud C, Bonichon F, Dejax C, Toubert ME, Leboulleux S, Ricard M, Benhamou E; Tumeurs de la Thyroïde Refractaires Network for the Essai Stimulation Ablation Equivalence Trial. Strategies of radioiodine ablation in patients with low-risk thyroid cancer. N Engl J Med. 2012;366:1663-1673.

### A2 Thyroid disorders and breast cancer

#### Peter Smyth^1,2^

##### ^1^University College Dublin, Dublin, Republic of Ireland; ^2^National University of Ireland, Galway, Republic of Ireland

In 1896 Beatson used thyroid extract accompanied by oophorectomy to treat breast cancer and thereby started a search for an association between thyroid disorders and breast cancer which continues to this day. However despite extensive studies frustratingly little evidence of a causative link has emerged. Many reports have associated breast cancer with hyperthyroidism, hypothyroidism, iodine deficiency and nontoxic goitre with hypothyroidism being the predominant breast related thyroid disorder [1,2]. This is not a universal finding as many reports fail to detect an association between the two disorders [3]. One area where there is some, although far from universal agreement, is the finding of an increased prevalence of antithyroid autoantibodies in breast cancer patients compared to controls [4]. This report reviews published evidence for the coincidence of thyroid disorders and breast cancer and highlights some areas which might benefit from further exploration. Studies in Ireland over a 20 year period (1990-2010) revealed a preponderance of both hypothyroidism and nontoxic goitre in patients with breast cancer compared to age matched controls [5]. Similarly TPO.Ab ± Tg.Ab were significantly greater in patients with breast cancer (30.5%) or benign breast disease (fibroadenoma 28.7%) compared to age matched controls (15.0% and 13.7% respectively) [6]. A previous report in an Irish breast cancer population showed that the presence of TPO.Ab and increased ultrasound measured thyroid volume was associated with improved outcome in breast cancer and was supported by studies that women with high levels of TPO.Ab had a lower risk of breast cancer with particularly better outcome in advanced breast cancers [7,8]. TPO has been shown to be expressed in breast cancer and it has been suggested that the expression of TPO in breast tissues may explain the protective role of TPO.Ab in breast cancer [9]. In addition to the association of thyroid disorders with breast cancer, it has been long suggested that a link between the two disorders is their common ability to concentrate iodide [10,11]. The demonstration that breast tissue expressed the iodide transporter NIS and that tissue iodine were found to be significantly lower in malignant compared to benign breast tissue [12] supported the suggestion that defects in iodine transport may have a role in breast cancer pathogenesis [13,14].

In an attempt to elucidate if the presence of TPO.Ab in breast cancer was an expression of a generalised immune response to the cancer or a true indication of autoimmune thyroid disease the author explored the relevance as to the significance of detectable rather than elevated levels of TPO.Ab in breast cancer [15,16]. Examining serum TPO.Ab concentration and its relationship to TSH in patients with postmenopausal breast cancer compared to postmenopausal controls it emerged that there was an association, not only between elevated TPO.Ab (>20.0 kIU/l) and serum TSH but that this association also applied to those patients with breast cancer who had detectable TPOAb (>2.0 < 20.0 kIU/l). Both TPO.Ab positivity and detectability were increased in breast cancer patients, even when TSH was within the upper 50% of the reference range (2.1-4.0 mU/l). The explanation for increased TPO.Ab prevalence in breast cancer is unknown but on the basis of serum TSH distribution, it may be associated with a subtle thyroid dysfunction. These observations lead the author to conclude that the association between thyroid disorders and breast cancer is based on more than chance. Coexisting autoimmune thyroid disease is the most likely candidate but it remains to be established if this observation can offer a therapeutic opportunity.

**Declarations**

**Ethics approval and consent to participate**

Not applicable

**Consent for publication**

Not applicable

**Availability of data and materials**

Not applicable

**Competing interests**

The author declares no conflict of interests.

**Funding**

Not applicable

**Peer review**

This short paper underwent the journal’s standard peer review process for supplements.

**References**

1. Goldman ME. Thyroid diseases and breast cancer. Epidemiol Rev. 1990;12:16-28.

2. Smyth PPA. The thyroid and breast cancer: a significant association? [Editorial] Ann Med. 1997;29:189-91.

3. Sarlis NJ, Gourgiotis L, Pucino F, Tolis GJ. Lack of association between Hashimoto thyroiditis and breast cancer: A quantitative research synthesis. Hormones 2002;1:35-41.

4. Smyth PP. Autoimmune thyroid disease and breast cancer: a chance association? J Endocrinol Invest. 2000;23:42-43.

5. Smyth PPA, Smith DF, McDermott P, Murray J, Geraghty JG, O'Higgins NJ. A direct relationship between thyroid enlargement and breast cancer. J Clin Endocrinol Metab. 1996 81:937–941.

6. Smyth PPA , Shering S, Kilbane MT, Murray MJ, Mc Dermott EWM, Smith DF, O'Higgins NJ. Serum thyroid peroxidase autoantibodies, thyroid volume and outcome in breast cancer. J Clin Endocrinol Metab. 1998;83:2711-2716.

7. Tosovic A1, Becker C, Bondeson AG, Bondeson L, Ericsson UB, Malm J, Manjer J. Prospectively measured thyroid hormones and thyroid peroxidase antibodies in relation to breast cancer risk. Int J Cancer 2012;131:2126-2133.

8. Kim SS1, Kim IJ, Kim SJ, Lee JY, Bae YT, Jeon YK, Kim BH, Kim YK. Incidental diffuse thyroid 18 F-FDG uptake related to autoimmune thyroiditis may be a favorable prognostic factor in advanced breast cancer. J Nucl Med. 2012;53:1855-1862.

9. Muller I, Giani C, Zhang L, Grennan-Jones FA, Fiore E, Belardi V, Rosellini V, Funel N, Campani D, Giustarini E, Lewis MD, Bakhsh AD, Roncella M, Ghilli M, Vitti P, Dayan CM, Ludgate ME. Does thyroid peroxidase provide an antigenic link between thyroid autoimmunity and breast cancer? Int J Cancer 2014;134:1706-14.

10. Eskin BA, Grotkowski CE, Connolly CP, Ghent WR. Different tissue responses for iodine and iodide in rat thyroid and mammary glands. Biol Trace Element Res 1995;49:9-19.

11. Venturi S. Is there a role for iodine in breast diseases? Breast 2001;10:379-382.

12. Kilbane MT, Ajjan RA, Weetman AP, Dwyer R, McDermott EWM, O'Higgins NJ, Smyth PPA. Tissue iodine content and serum mediated ^125^I uptake blocking activity in breast cancer. J Clin Endocrinol Metab. 2000;85:1245-1250.

13. Smyth PPA. The thyroid, iodine and breast cancer. Breast Cancer Res. 2003;5:235-238.

14. Aceves C, Anguiano B, Delgado G. Is iodine a gatekeeper of the integrity of the mammary gland? J Mammary Gland Biol Neoplasia. 2005;10:189-196.

15. Tozzoli R, Giavarina D, Villalta D, Soffiati G, Bizzaro N. Definition of reference limits for autoantibodies to thyroid peroxidase and thyroglobulin in a large population of outpatients using an indirect method based on current data. Arch Pathol Lab Med. 2008;132:1924-1928.

16. Feldt-Rasmussen U, Verburg FA, Luster M, Cupini C, Chiovato L, Duntas L, Elisei R, Rimmele H, Seregni E, Smit JW, Theimer C, Giovanella L. Thyroglobulin autoantibodies as surrogate biomarkers in the management of patients with differentiated thyroid carcinoma. Curr Med Chem. 2014;21:3687-3692.

### A3 New thyroid hormone metabolites: biosynthesis, action, and clinical relevance

#### Josef Köhrle

##### Institut für Experimentelle Endokrinologie, Charité-Universitätsmedizin Berlin, Berlin, Germany

Classical action of thyroid hormones (TH) on foetal and postnatal development, especially of the brain, on growth, energy metabolism, metabolic rate as well as on many anabolic and catabolic pathways is thought to be exerted via T3–dependent modulation of the function of various nuclear, mitochondrial and cytosolic isoforms and isotypes of T3 receptors (TR). These ligand-dependent transcription factors eventually modulate expression of T3-regulated genes. L-thyroxine (T4), the main product secreted by the human thyroid gland, is considered as a “prohormone” devoid of inherent biological activity with exception of its rapid activation of anb3 integrin receptor signalling at the cell membrane. These integrin receptors also respond to the T4 metabolite Tetrac, which is found in blood in concentrations similar to those of T3. Reverse-T3, circulating in regulated manner in the blood is also regarded as an inactive TH metabolite but may act in guidance and regulation of neuronal migration during CNS development.

Recently, marked “thyromimetic” effects have been reported for 3,5-T2, a *“hot”* metabolite putatively formed from T3. Via several mitochondrial targets 3,5-T2 rapidly stimulates mitochondrial function and oxygen consumption, “cleans” steatotic livers from lipid accumulation, improves hyperlipidemia and hypercholesterolemia in rodent models and acts on skeletal muscle. 3,5-T2 has been reported to lack thyromimetic action on cardiac function and the hypothalamus-pituitary-thyroid (HPT) axis in various rat animal experimental models [1]. The in vivo pathways of 3,5-T2 formation are still elusive, though PTU inhibition of its formation and action indicates a contribution of PTU-sensitive type 1 5’-deiodinase in its biosynthesis. However, these alleged “beneficial” 3,5-T2 effects are still controversial as several mouse and rat experimental studies indicate dose-dependent adverse effects on the heart (increased weight, hypertrophy, altered gene expression, fibrotic signs), intermediary metabolism and suppression of the HPT axis. Therefore, the use of this compound, freely available by internet or as OTC preparation in the body-building, doping and wellness scene should be strongly discouraged and physicians need to be aware of potential abuse of 3,5-T2 by their patients showing ‘abnormal’ thyroid function tests. A chemiluminescence immunoassay (CLIA) has recently been developed for 3,5-T2 [2] and its first application in clinical studies revealed elevated 3,5-T2 serum concentrations under several conditions associated with the “low-T3 syndrome” or non-thyroidal illness.

3-iodothyronamine (3T1AM) is a decarboxylated *“cool”* TH metabolite formed via combined deiodination and decarboxylation [3,4]. All three deiodinases can handle thyronamines and ornithine decarboxylase (ODC) as well as gastrointestinal mucosa have been proposed to contribute to 3T1AM biosynthesis. 3T1AM, detected by CLIA in human serum in low nanomolar concentrations has a long half-life probably due to its binding to apolipoprotein B100. Serum 3T1AM concentrations in patients on T4 replacement therapy are higher than in healthy individuals and several correlations to metabolic parameters have been suggested. In animal models administration of pharmacological doses of 3T1AM rapidly decrease body temperature, increase glucagon and glucose but reduce insulin serum concentrations and shift metabolism from carbohydrate to lipid oxidation. Several behavioural effects on altered learning, pain sensation and transmitter action have been described [5]. 3T1AM acts via several receptor systems such as trace amine-associated receptors (TAAR), transient thermosensitive receptor potential melastatin 8 (TRPM8) channel and α-2A-adrenergic receptor. Further CNS, metabolic and cardiac effects have been reported for N-acetylated and acetic acid metabolites of 3T1AM.

Supported by grants of the Deutsche Forschungsgemeinschaft DFG SPP 1629 ThyroidTransAct.

**Declarations**

**Ethics approval and consent to participate**

Not applicable

**Consent for publication**

Not applicable

**Availability of data and materials**

Not applicable

**Competing interests**

The author declares no conflict of interests.

**Funding**

Not applicable

**Peer review**

This short paper underwent the journal’s standard peer review process for supplements.

**References**

1. Goglia F. The effects of 3,5-diiodothyronine on energy balance. Front Physiol. 2015;5:528.

2. Lehmphul I, Brabant G, Wallaschofski H, Ruchala M, Strasburger CJ, Köhrle J, Wu Z. Detection of 3,5-diiodothyronine in sera of patients with altered thyroid status using a new monoclonal antibody-based chemiluminescence immunoassay. Thyroid 2014;24:1350-1360.

3. Piehl S, Hoefig CS, Scanlan TS, Köhrle J. Thyronamines – past, present, and future. Endocr Rev. 2011;32:64-80.

4. Scanlan TS. Endogenous 3-iodothyronamine (T1AM): more than we bargained for. J Clin Endocrinol Metab. 2011;96:1674-1674.

5. Zucchi R. Accorroni A, Chiellini G. Update on 3-iodothyronamine and its neurological and metabolic actions. Front Physiol. 2014;5:402.

## Other presentations: short papers and extended abstracts

### A4 Short-stay surgical goitre treatment. Possibilities and conditions

#### Ryszard Anielski^1,2^, Marian Slowiaczek^1,^, Pawel Orlicki^1,2,^

##### ^1^Department of Short-stay Surgery, St. John Grande Hospital of Brothers Hospitallers, Cracow, Poland; ^2^Department of Catastrophe Medicine and Emergency Medicine, Chair of Anesthesiology and Intensive Care, Jagiellonian University, Medical College, Cracow, Poland

###### **Correspondence**: Ryszard Anielski

**Background**

Operations have their recognised place in the treatment of goitre. The indications for operative treatment have been the same for many years. Advances in medicine and changes in surgical approach have both influenced the development of minimally invasive techniques. These have also affected the duration of hospital stay, shortening it significantly, and resulting in a reduction in the real financial costs of the medical services provided.

**Aims**

The aim of study was to evaluate the use of short-stay (one-day) surgery in patients with thyroid disorders requiring surgical treatment.

**Material and methods**

A prospective analysis was performed of two groups of operated patients with a variety of thyroid conditions. One group consisted of patients treated at the Short-stay Surgical Department of the St. John Grande Hospital of Brothers Hospitallers, in Cracow. The second consisted of the material of 500 patients operated on conventionally in the Department of Endocrine Surgery of the Third Chair of General Surgery, Jagiellonian University Medical College in Cracow by the same surgeons in the years 2007-2008. The groups of patients were then compared and analysed statistically. Statistical analysis involved comparing the groups with respect to specific diagnostic criteria, using the chi-square test and Fisher's exact test for categorized discrete variables, and the Mann-Whitney U and Kruskal-Wallis tests for continuous variables. The Student-t test was employed to calculate probability and confidence interval.

**Results**

Between 2010 and 2015, 102 operations for goitre were performed in the Department of Short-stay Surgery of the St. John Grande Hospital of Brothers Hospitallers, Cracow. The comparison group consisted of the material of 500 patients operated for thyroid disease in the Department of Endocrine Surgery JU CM (DEPT) in the years 2007 – 2008. The groups were similar with respect to age and sex. The duration of post-operative stay in the first group (HOSPITAL) was 1.9 days ± 0.3, as compared with 3.8 days ± 1.36 in the second group (DEPT) (p < 0.001) (Table 1).

In assessing patients for potential short-stay surgery, those for overactive goitre were less frequently qualified than those with thyroid inflammation (p < 0.001). There were no significant differences in the other types of goitre (Table 2).

We must note that the cancers were at a less advanced stage in patients operated for thyroid cancer under the short-stay regime, than those in the second group. The extent of the operation, and operative technique were similar between the two groups studied (Table 3).

Complications arising were also compared, and these were less frequent in operative patients in the first (hospital) group (7.8% vs 17.8%) (p = 0.012).

**Conclusion**

Short-stay surgical treatment of thyroid conditions in specialist centres is possible and is as safe as conventional-term treatment, with no increase in the frequency of postoperative complications.

**Declarations**

**Ethics approval and consent to participate**

No, becuase there is a retrospective analysis.

**Consent for publication**

Not applicable

**Availability of data and materials**

Available from the corresponding author on reasonable request.

**Competing interests**

The authors declare no conflict of interests.

**Funding**

Not applicable

**Authors' contributions**

R.Anielski: concept of the study, data collection and analysis, writing of the manuscript

M.Słowiaczek: data collection, writing of the manuscript, discussion and critical revision.

P.Orlicki: data collection, substantive evaluation of the manuscript and discussion.

**Peer review**

This extended abstract underwent the journal’s standard peer review process for supplements.

**Table 1 (abstract A4).** Population characteristics

Data		HOSPITAL	DEPT	*p*
SEX	F	88.2%	87.2%	0.077
	M	11.8%	12.8%	
Mean age (years)		51 ± 16	53 ± 13	NS
Duration of stay (days)		1.9 ± 0.3	3.8 ± 1.4	<0.001

**Table 2 (abstract A4).** Clinical diagnosis of patients

Clinical Diagnosis	HOSPITAL		DEPT		*p*
	n	%	n	%	
Cancer (primary op.)	12	11.8	57	11.4	NS
Cancer (radicalisation)	1	1	4	0.8	NS
Nodular goitre	69	67.6	306	61.1	NS
Toxic nodular goitre	5	4.9	105	21.1	<0.001
Graves’ disease	0	0	9	1.8	NS
Inflammation of thyroid	13	12.7	1	0.2	<0.001
Recurrent goitre	2	2	18	3.6	NS
TOTAL	102	100	500	100

**Table 3 (abstract A4).** Type of surgical procedure

Type of operations	HOSPITAL		DEPT		*p*
	n	%	n	%	
Total thyroidectomy with dissection of lymph node central neck compartment	9	8.8	12	2.4	0.004
Total thyroidectomy	68	66.7	283	56.6	0.06
Subtotal thyroidectomy	6	5.9	130	26	<0.001
Bilateral subtotal thyroidectomy	0	0	6	1,2	NS
Lobectomy	18	17.6	63	12.6	NS
Secondary thyroid operations	1	1	3	0.6	NS
Palliative thyroid resection	0	0	3	0.6	NS
TOTAL	102	100	500	100	

### A5 How to prepare the patient for radioiodine treatment?

#### Rafal Czepczynski

##### Department of Endocrinology, Metabolism and Internal Diseases, Poznan University of Medical Sciences, Poznan, Poland

Radioiodine therapy (RIT) is an important method of treatment of hyperthyroidism. Two different approaches to RIT are represented with regard to dosage of radioiodine (^131^I): individual dose calculation based on a detailed dosimetry and the administration of fixed doses of ^131^I aimed at the hypothyroidism as the final outcome. As shown by the statistics on huge numbers of patients followed for decades after RIT, even using meticulous calculation of the activity of therapeutic ^131^I, hypothyroidism is very common – it is found in approx. 30% of patients with toxic nodular goitre and even in 80% of patients with Graves' disease. The risk of hypothyroidism increases with each passing year after RIT at a rate of approximately 3-5% per year.

RIT effect depends on many unmodifiable factors, for example thyroid mass, clinical form of hyperthyroidism, hormonal status etc. Several studies have demonstrated reduced efficacy of RIT in patients with lower TSH concentrations, higher thyroxine concentrations and higher TRAb titres. There is, however, a number of factors that can be modified to obtain a better result of RIT. The efficacy of RIT is primarily dependent on the kinetics of iodine in the thyroid gland. It can be followed using dosimetric measurements. A spot measurement of thyroid iodine uptake after 24 h (IU24) may not reflect the actual exposure of the gland to beta radiation. High values of IU24 are associated with poorer treatment effect, as these patients present a rapid iodine turnover and ^131^I is retained in the thyroid for a relatively short time. Iodine kinetics can be improved by low-iodine diet. Reducing the supply of iodine by the reduction of food intake based on fish, seafood and marine plants, as well as dairy products (milk, cheese) for 1-2 weeks, can increase the uptake of iodine by 20-30%. Longer elimination of iodine is not recommended because of possible side effects, e.g. hyponatremia. In the case of very low IU24 values, lithium salts can improve the effect of RIT. Although lithium is not responsible for the increase of iodine uptake by the thyroid gland, it does increase the retention time of iodine in the thyroid. The use of approx. 1 g of lithium carbonate per day for 5-14 days resulted in an increase of absorbed dose and a reduction of cases with the therapy failure. More and more often rhTSH is used to stimulate iodine uptake. A single injection of rhTSH causes usually 2-4-fold increase in the ^131^I uptake. Due to stimulation of the thyroid hormone release, this method is mainly used in the treatment of non-toxic nodular goitre. Stimulation with rhTSH frequently causes side effects like pain at the base of the neck and temporary increase of the thyroid volume.

Antithyroid drugs used before, during and after RIT adversely affect the final outcome of the treatment (number of patients achieving euthyroid or hypothyroid status after RIT is lower). This is connected with their impact on iodine uptake, their immunomodulatory action and radioprotective effect in the thyrocyte. This effect is more pronounced in the case of propylthiouracil than methimazole. Therefore, while preparing the patient to RIT, following expert recommendations should be followed: refrain from the use of these drugs in case of slight hyperthyroidism, in more severe cases prefer methimazole over PTU and withdraw the drug for 2-7 days (methimazole) or 2-3 weeks (propylthiouracil) before RIT. If necessary, an antithyroid drug can be resumed after RIT, optimally approx. 1 week after the administration of 131I.

Corticosteroids, amiodarone and iodine contrast agents should be named among the drugs that adversely affect the effect of RIT. Through their cytoprotective and immunosuppressive effect, corticosteroids reduce the radiosensitivity of the thyroid cells. Therefore, for the prevention of Graves orbitopathy, corticosteroids should be introduced a few days after RIT. Although the contrast agents contain large amounts of iodine (approx. 30 g of organic iodine and 5 mg of iodide in an average dose), urinary iodine excretion returns to normal as early as after 1-2 months after injection. Amiodarone treatment is regarded as an absolute contraindication to RIT. However, as demonstrated by retrospective studies in our centre, RIT used in an experimental setting in such patients even at very low IU24 led to normalization of amiodarone-induced hyperthyroidism in a few months.

RIT often leads to hypothyroidism. Nevertheless, in accordance with the ALARA rule, we should strive to achieve a therapeutic effect using the lowest possible radionuclide activity. Thus, taking the factors described above into account while preparing the patient to RIT will allow to optimize the treatment effect and to reduce the exposure of the patient and the environment (including ourselves) to ionizing radiation.

**Declarations**

**Ethics approval and consent to participate**

Not applicable

**Consent for publication**

Not applicable

**Availability of data and materials**

Not applicable

**Competing interests**

The author declares no conflict of interests.

**Funding**

Not applicable

**Peer review**

This short paper underwent the journal’s standard peer review process for supplements.

### A6 Management of mild thyroid orbitopathy

#### Jacek Daroszewski

##### Department of Endocrinology, Diabetes and Isotope Therapy, Medical University, Wroclaw, Poland

Thyroid orbitopathy (TO), the most frequent extrathyroidal manifestation of Graves’ disease (GD) occurs in approximately 2 per 10 000 population per year and in 25%–60% of patients with GD in clinically relevant form. Progression of mild to moderate-to-severe TO may occur in up to 15% of subjects and no predictors are available to predict worsening at a first clinical examination. Therefore the management of mild TO and prevention of the aggravation is one of the most common problem in practical endocrinology.

Mild TO is usually diagnosed basing on soft tissue involvement (conjunctival and eyelid oedema and congestion) and proptosis < 3 mm above the upper limit. However it must be noted, that eye muscle involvement may be often underestimated due to low sensitivity of generally used motility tests. Despite often discrete manifestations at clinical examination diplopia is the principal symptom influencing patients’ quality of life (QoL) and his work ability. Moreover, as subjective factor as the impact of symptoms on patient’s everyday life and work must be considered. Therefore in big proportion of patient the grading of TO and a subsequent decision on implementation the immunosuppressive treatment is based on patient’s self-assessment.

Strict metabolic control with the avoidance of hypothyroidism is a principal element of the treatment of thyrotoxicosis in GD patients. Changes in thyroid status may negatively influence TO. Therefore, evaluation of thyroid function ought to be frequent (every 6–8 weeks or even more often) during the first phases of treatment (or after changing the daily dose of the ATD) and periodical (every 3–4 months) thereafter.

Treatment methods for hyperthyroidism [ATDs, radioiodine (RAI), thyroidectomy] *per se* seem to be capable to affect the course of TO and the choice of method is still a challenging dilemma. Randomized trials showed that TO grew more often after RAI than after thyroidectomy or ATDs .

The influence of RAI on TO are uncertain, due to the limited number of controlled studies. Steroid prophylaxis is recommended in patients on RAI treatment, if mild and active TO pre-exists. Because TO may also appear for the first time after RAI treatment, it is generally worth wide to consider the pros and cons of steroid use also in GD patients with risk factors as male gander, age > 60 yrs, smoking and high TRab titre. Steroid prophylaxis can be performed with very low doses of prednisone (0.2 mg/kg body weight), given 1 day after RAI therapy, gradually tapered down and stopped after 6 weeks. In subject with risk factors for the RAI-associated TO progression, doubled both doses of prednisone and time of treatment should be implemented.

Tabaco smoking markedly increases the risk of developing TO and aggravate it is course. Smokers are 4-fold more prone to develop eye symptoms and as much as 27-times more often experience the severe stage. Smokers respond less well to treatment with steroids or radiotherapy then non-smokers (15% vs. 85%). The efficacy of helping patients stop smoking is very low: opportunistic advice from doctor only 2%. Therefore all GD patient should be honestly informed about the risk and nicotine replacement therapy may be considered.

GD subjects are at high risk of dry eye symptom due to mechanical factors, hormonal disturbance, autoimmunological process and side effects of commonly used drugs. Orbital discomfort (at rest or with gaze) must be distinguished from ocular surface irritation from exposure. It may be also hypothesized the inflammation at the eye surface *via* proinflammatory cytokines may induce inflammation in retrobulbar space. Therefore all subjects with OT and may be the majority of GD patients, should be advised to consistently use local measures. Artificial tear drops or gels (administered 4-6 times daily – in the morning, by meals and at bed time) and lubricant ointments prior to slipping can reduce surface symptoms and improve quality of life. The use of preservative-free drops can help to avoid allergic reactions, epithelial toxicity and conjunctival inflammation by preservatives and particularly benzalkonium chloride.

Non-ophthalmologists often forget, that such simple solutions can have a significant impact on TO symptoms.

Antioxidant therapy in TO has been used for ca. 30 years but reported results are extremely divergent. Hoverer randomized, placebo control study conducted by EUGOGO showed selenium (but not pentoxyfylline) treatment within 6 months induced significant improvement in eye status and QoL during 12 month follow up. Therefore selenium supplementation (200 μg daily) is recommended for patients with mild TO and may be advised for GD subjects free of TO but with risk factors.

**Declarations**

**Ethics approval and consent to participate**

Not applicable

**Consent for publication**

Not applicable

**Availability of data and materials**

Not applicable

**Competing interests**

The author declares no conflict of interests.

**Funding**

Not applicable

**Peer review**

This short paper underwent the journal’s standard peer review process for supplements.

### A7 Management of the subclinical hypothyroidism (SH) in pregnancy – are the current guidelines expected to be changed?

#### Alicja Hubalewska-Dydejczyk

##### Chair and Department of Endocrinology, Jagiellonian University, Medical College, Cracow, Poland

Thyroid dysfunctions in pregnancy are relatively common and they have a negative impact on the health of the mother and her offspring. The physiological changes during pregnancy influence the thyroid function, thus the existing guidelines recommend the necessity to prepare the trimester-specific thyroid hormone reference ranges for each medical centre and analytical method used. In case of a lack of such TSH reference ranges the following ones are generally accepted: I trim: 0.1-2.5 mU/L, II and III trim. 0.2-3.0 (ATA, ES) – 3.5 mU/L (ETA).

While discussing the thyroid illness in pregnant women, it has to be underlined that despite the well-informed guidelines and expert work there are areas of substantial uncertainty. Among others the most important questions are: how to define the SH during pregnancy, can the maternal SH adversely affect the foetus and the mother and how to assess/balance the risk and benefit of any intervention. The endpoint of the clinical studies are the pregnancy complications and foetal cognitive development as possible, well-known hypothyroidism-related disturbances. The dilemmas in relation to the normal TSH reference range followed by the SH definition in pregnancy result from the differences caused by variations in assays, population-specific factors such as ethnicity, dietary habits, iodine status, BMI. These dilemmas can also be caused by the fact that the even slight abnormalities in thyroid function are potentially unfavourable factors associated with child outcome. Maternal thyroid hormones (TH) are the only source of TH for foetus especially in the first half of pregnancy.

What is normal? – this is the most frequent question. According to the International Federation of Clinical Chemistry the reference intervals have to be based on the 2.5^th^ and 97.5^th^ percentile of the respective population with an optimal iodine intake in case of thyroid hormone ranges assessment. In meta-analysis based on the 14 well-performed studies (in which TSH and FT4 were calculated in accordance with the international guidelines and the sufficient number of pregnant women with negative thyroid antibodies were enrolled to the studies), Medici et al. [1] showed that there are essential differences in upper TSH reference value during early pregnancy worldwide and in 90% of cases TSH upper limit value exceeds the recommended TSH level (2.63-4.68 mU/L). The results of the Generation R study [2], a large cohort study, suggest that the diagnosis of the SH should be undertaken when TSH >4.04 mU/l and normal FT4 (10.4-22.0 pmol/L). Bestwick et al. [3] suggest using multiple of the median (MoM) values to overcome the limitation connected with TSH and TH assessment during pregnancy. TSH and FT4 (MoMs) significantly reduced the difference between UK and Italian samples compared with conventional units, which were also confirmed by Medici et al. in the above, cited work in different populations [1].

In the face of the above facts, there are critical questions whether our pregnant women are treated in euthyroidism and whether the treatment of the euthyroid pregnant women has a negative effect on pregnancy course and foetus.

There are some new clinical data confirming that even subclinical thyroid dysfunction in pregnancy increases the risk of mother and child adverse outcomes. Among others, Liu et al. [4], in prospective cohort study of 3315 women screened, showed that the women with SCH were at an increased risk of miscarriage between four and eight gestational weeks but statistical significance was achieved only if TSH ≥5.22 mU/L. Women with a combination of SCH and TAI were found to were at the highest risk of miscarriage and, for that, at earlier gestational ages.

In conclusion, it should be stated, that the current available data concerning the upper TSH limit value in pregnancy, safe for mother and her offspring, could probably be higher than previously thought. It requires further clinical studies, however, thyroid related research in pregnant population is difficult to conduct.

**Declarations**

**Ethics approval and consent to participate**

Not applicable

**Consent for publication**

Not applicable

**Availability of data and materials**

Not applicable

**Competing interests**

The author declares no conflict of interests.

**Funding**

Not applicable

**Peer review**

This short paper underwent the journal’s standard peer review process for supplements.

**References**

1. Medici M, Korevaar TI, Visser WE, Visser TJ, Peeters RP. Thyroid function in pregnancy: what is normal? Clinical Chem. 2015;61:704-713.

2. Medici M, de Rijke YB, Peeters RP, Visser W, de Muinck Keizer-Schrama SM, Jaddoe VV, Hofman A, Hooijkaas H, Steegers EA, Tiemeier H, Bongers-Schokking JJ, Visser TJ. Maternal early pregnancy and newborn thyroid hormone parameters: the Generation R study. J Clin Endocrinol Metab. 2012;97:646-652.

3. Bestwick JP, John R, Maina A, Guaraldo V, Joomun M, Wald NJ, Lazarus JH. Thyroid stimulating hormone and free thyroxine in pregnancy: expressing concentrations as multiples of the median (MoMs). Clin Chim Acta 2014;430:33-37.

4. Liu H, Shan Z, Li C, Mao J, Xie X, Wang W, Fan C, Wang H, Zhang H, Han C, Wang X, Liu X, Fan Y, Bao S, Teng W. Maternal subclinical hypothyroidism, thyroid autoimmunity, and the risk of miscarriage: a prospective cohort study. Thyroid 2014; 24:1642-1649.

### A8 Delayed risk stratification as the indicator of treatment and follow-up in thyroid cancer patients

#### Aldona Kowalska

##### Endocrinology Department, Holycross Cancer Centre, Kielce, Poland

There has been a dynamic increase in detectability of differentiated thyroid cancer (DTC) in recent years. This increase is mainly due to detection of small foci of papillary carcinoma, which is characterized by very good prognosis. Increased morbidity does not result in increased mortality. Currently the management of the DTC patient dictates a lifelong oncologic surveillance due to recurrences or distant metastases observed even many years after the onset of the disease. Diagnostics and treatment can be a significant burden for the patients. Thyroid surgery involves the risk of hypoparathyroidism and damage of the recurrent laryngeal nerve. The frequency of surgical complications depends on surgeon's experience and scope of the surgery. The risk of postsurgical hypoparathyroidism significantly increases with the central compartment lymph node dissection and when the surgery is performed by an inexperienced surgeon (less than 10 surgeries a year). Another burden for the patient is using the ^131^I therapy. It has been indicated that using an isotope with the activity above 100 mCi increases the risk of leukaemia. Another danger is the increased risk of salivary gland cancer or fertility impairment. The way to avoid the above complications is using the ^131^I with possibly lowest activity and stimulating the ^131^I uptake with recombinant TSH instead of LT4 withdrawal. It has also been indicated that using Thyrogen decreases the ^131^I radiotoxicity by 30-50%, while maintaining the high effectiveness of ablation. Discontinuation of the ^131^I therapy is also essential in groups of very low recurrence risk, where benefits of such therapy have not been indicated. The usage of suppressive LT4 doses increases the risk of atrial fibrillation and has an influence on bone mineral density decrease in postmenopausal women. Periodic oncologic follow-up which include stimulated Tg measurement or a whole–body scan, what is further associated with physical and psychological burden. Adjusting the treatment and monitoring the disease is necessary in case of poor disease outcome. There are ongoing studies on several risk stratification systems. Seventh edition of the TNM system according to AJCC/UICC is commonly used. This system correlates very well with the risk of fatal outcome, however, it does not allow a good differentiation of high risk recurrent/persistent disease patients. Using the system is recommended worldwide, as it allows keeping the epidemiological registers and monitoring the changes in DTC epidemiology. Stratification systems recommended by ATA and ETA are also used to assess the risk of disease recurrence, taking into account assessment of the histological type and surgical radicality of the procedure. In 2015, new ATA recommendations advise taking into account the number and extent of the lymph node involvement (>5 and the size of metastasis in a node >0.2 cm and clinically identified nodes) as factors which affect the prognosis. Other factors which affect the prognosis are the degree of the extrathyroidal extension (microscopic or extensive) and the degree of vascular invasion in follicular thyroid cancer. Attempts are being made to use molecular factors (*BRAF, TERT*) as prognostic elements, but the data concerning this subject is inconclusive. In 2010, Tuttle et al. suggested a delayed risk stratification system (DRS), applied 2 years after finishing the initial treatment. The system assumes effectiveness of the therapy as a predictor. Patients, who respond very well to the treatment (Tg/LT4 < 0.2, Tg/stimulated <2.0, no evidence of disease at ultrasound examination and a whole-body scan), constitute the low-risk group. Whereas, patients with biochemically persistent disease (Tg/LT4 > 1.0, Tg/stimulated >10.0, increasing anti-Tg serum) or structurally persistent (disease seen in imaging and physical examinations) constitute the high-risk group. The group with ambiguous response is composed of patients with serum Tg/stimulated >2.0 < 10, stable or decreasing anti-Tg levels, nonspecific changes in imaging. Studies carried out by several teams confirm high effectiveness of the above stratification system in predicting the outcome. It is recommended to use the delayed risk stratification system to personalize the LT4 treatment by adjusting the suppression level to the risk as well as by modifying the intensity and method of the oncologic follow-up. Using the DRS system will allow many patients to avoid negative outcomes of the LT4 suppressive therapy as well as mentally and physically burdensome periodic follow-up of possible disease remission. At the same time, it will bring significant savings for the health care budget.

**Declarations**

**Ethics approval and consent to participate**

Not applicable

**Consent for publication**

Not applicable

**Availability of data and materials**

Not applicable

**Competing interests**

The author declares no conflict of interests.

**Funding**

Not applicable

**Peer review**

This short paper underwent the journal’s standard peer review process for supplements.

### A9 Influence of vitamin D3 deficiency on body mass and TSH level in patients with Hashimoto’s thyroiditis

#### Maria Kurowska, Joanna Malicka, Anna Oszywa-Chabros, Agnieszka Zwolak, Jerzy S. Tarach

##### Chair and Department of Endocrinology, Medical University of Lublin, Lublin, Poland

###### **Correspondence:** Jerzy S. Tarach

**Background**

One billion people worldwide are characterized by vitamin D deficiency. Vitamin D deficiency is also prevalent in subjects with Hashimoto`s thyroiditis. It was proved that serum 25(OH)D levels in these patients are lower than in healthy individuals. This observation suggests that vitamin D deficiency may play an important role in the development of thyroiditis. Several clinical trials have shown that supplementation of vitamin D may reduce the incidence of rheumatoid arthritis, multiple sclerosis and type 1 diabetes. In experimental studies it was proved that 1,25(OH)_2_D_3_ may prevent Hashimoto`s disease, however the mechanisms of this impact has so far not been explained. It was also reported that administration of vitamin D together with anti-thyroid drugs or thyroid hormones suppresses the autoimmune reaction and reduces serum levels of thyroid autoantibodies. The discovery of the presence of vitamin D receptors in the thyroid gland probably indicates that vitamin D plays a role in regulation of thyroid function. The association of hypothyroidism with higher body mass is commonly known. Also the state of vitamin D supply is inversely correlated with obesity.

**Aims**

To determine whether there is a correlation between the concentration of 25(OH)D_3_, body weight and TSH level in patients with Hashimoto's thyroiditis.

**Materials and methods**

Covered 65 patients [55 F; 10 M] aged 20-86 years [mean 50.5 ± 20.3], hospitalized in our department between 2013-2015. Analysis of clinical picture and laboratory studies. Serum TSH levels were measured with CLIA and 25(OH)D_3_ with ECLIA assays.

**Results**

Vitamin D_3_ deficiency <20 ng/mL was found in 45 [40 F; 5 M] = 69.2% of patients. In 15.4% [n = 10], vitamin D_3_ concentrations ranged from 20 to 30 ng/mL [hypovitaminosis], while in another 15.4% vitamin D_3_ levels were within the normal range [>30 ng/mL]. Depending on the concentration of 25(OH)D_3_ the patients were divided into 4 groups: group 1 covered 10 people [6 F; 4 M] with 25(OH)D_3_ concentration >30 ng/mL [average value 41.2 ± 9.2]; group 2 – 10 patients [9 F; 1 M] with 25(OH)D_3_ level between 20 and 30 ng/mL [average 22.5 ± 2.6]; group 3 – 22 people [20 F; 2 M] with 25(OH)D_3_ value between 10-20 ng/mL [mean 14.4 ± 2.8]; group 4 – 23 patients [19 F; 4 M] with 25(OH)D_3_ concentration <10 ng/mL [mean 6.8 ± 1.9]. BMI for groups 1 to 4 was respectively: 25.0 ± 5.6 kg/m^2^; 23.5 ± 4.4 kg/m^2^; 27.1 ± 6.1 kg/m^2^ and 27.6 ± 4.9 kg/m^2^. BMI in groups 3 and 4 was significantly higher (p <0.001) than in group 1. Mean TSH levels were respectively: in group 1 – 1.4 ± 1.4 [range from 0.1 to 3.9] mIU/L; in group 2 – 1.5 ± 1.2 [0.1-4.2] mIU/L; in group 3 – 10.4 ± 22.9 [0.1-87.0] mIU/L; in group 4 – 3.6 ± 4.8 [0.73-19.3] mIU/L. TSH levels differed between group 1 and group 4 statistically significantly [p < 0.001]. Additionally, all patients were divided into two major groups: group 1 = 20 people [15 F; 5 M] with 25(OH)D_3_ > 20 ng/mL and group 2 = 45 patients [40 F; 5 M] with 25(OH)D3 < 20 ng/mL. Average 25(OH)D_3_ concentrations were respectively: 31.84 ± 11.65 ng/mL in group 1 and 11.05 ± 4.52 ng/mL in group 2 (p < 0.001), TSH levels: 1.47 ± 1.31 mIU/L and 6.9 ± 16.5 mIU/L (p < 0.001), and BMI: 24.27 ± 5.15 kg/m^2^ and 27.37 ± 5.48 kg/m^2^ (p < 0.001). The age of patients in both groups was respectively: 41.55 ± 19.24 and 54.69 ± 5.48 years (p < 0.001).

**Conclusions**

Patients with Hashimoto's thyroiditis and 25(OH)D_3_ concentrations below 20 ng/mL are characterized by higher body weight and higher TSH levels. Our results suggest that vitamin D_3_ supplementation may have a positive effect on the proper control of hypothyroidism and body weight in patients with Hashimoto's disease.

**Declarations**

**Authors' contributions**

MK, JST: study conceived and designed, manuscript composition, critical revision and supervision. JM, AOC, AZ took part in the care of the patients, collected data and performed statystical analyses, literature search and helped with manuscript preparation.

**Declarations**

**Ethics approval and consent to participate**

The reported study involved human participants and it meets the Ethical Principles of the Declaration of Helsinki as well as of the Ethical Committee of Lublin Medical University.

**Consent for publication**

Not applicable

**Availability of data and materials**

Available from the corresponding author on reasonable request.

**Competing interests**

The authors declare no conflict of interests.

**Funding**

Not applicable

**Peer review**

This extended abstract underwent the journal’s standard peer review process for supplements.

### A10 Complications of antithyroid drug use

#### Krzysztof C. Lewandowski

##### Department of Endocrinology and Metabolic Diseases, The Medical University of Lodz, Lodz, Poland

The aim of the lecture is to present a summary on main aspects related to complications resulting from the use of thionamides for treatment of thyrotoxicosis, including methimazole (MMI), carbimazole (CMZ) and propylothouracil (PTU). Though benign complications, such allergic rash, urticaria or pruritus are common, main issues related to adverse effects of antithyroid drugs include hepatotoxicity, birth defects as well as agranulocytosis.

Methimazole/carbimazole-induced hepatotoxicity usually develops in the first few weeks of drug consumption with an estimated incidence of 0.1-0.2%. Hepatic abnormalities associated with methimazole are typical of a cholestatic process. In contrast, PTU-induced hepatotoxicity is of hepatocellular type in most cases, and hence is considered to be more serious. The frequency of PTU-related severe liver damage is approximately 0.1% based on the available data [1, 2]. In rare instances PTU administration might be associated with development of ANCA-related vasculitis [3].

Up till recently PTU was considered the drug of choice during pregnancy, because of small risk of MMI/CMZ embryopathy (such as *aplasia cutis*), though these recommendations had been contested. Realization of consequences of PTU-induced hepatotoxicity, including liver failure [2] resulted in change of current recommendations, particularly pertaining to pregnancy. In particular, current recommendations (e.g. Thyroid 2011;21:1081–1125) advise administration of PTU only in the first trimester of pregnancy, with subsequent change into MMI/CBZ. Recent studies, however, have cast doubt on the validity of these recommendations. First of all, thyrotoxicosis *per se* seems to be associated with an increased risk of birth defects [4]. Furthermore, both MMI/CBZ and PTU seem to associated with a similar increase in the frequency of birth defects, though different type of defects seem to occur in women taking MMI/CBZ *versus* those taking PTU [4, 5]. There are also no available data showing any benefit of switching from MMI/CBZ to PTU in the first trimester of pregnancy in terms of a risk of birth defects [5]. Hence in the recent commentary [6], the British authors recommend against switching to PTU in the first trimester of pregnancy, and recommend continuation of the lowest possible doses of MMI/CBZ.

Agranulocytosis (and in rare cases even more severe complications such as pancytopaenia or aplastic anaemia) remains the most feared complication of thionamide use. It should be mentioned that mild leukopenia is commonly observed in thyrotoxicosis, particularly in Graves’ disease. Incidence of agranulocytosis is considered to be around 3-4 cases per 10000 patients. According to the recent large retrospective study from Japan [7], agranulocytosis developed within 90 days after starting ATD therapy (84.5% out of 754 cases of agranulocytosis), and more commonly in females. Methimazole dose administered at the onset of agranulocytosis was 25.2 ± 12.8 mg/day (mean ± SD).

There is, however, no universal agreement as to the best strategy to detect agranulocytosis, and in particular in relation to the usefulness of routine monitoring of white cell counts (WCC). There is a possibility that there are at least two patterns of development of agranulocytosis. The first, being much more common, involves a sudden autoimmune attack with rapid development of agranulocytosis, even within 24-48 hours. Indeed Nakamura et al. [7], demonstrated cases of agranulocytosis that developed suddenly, despite a normal WCC that had been observed even at one to two days prior to detection of agranulocytosis. Yet, there is also another pattern that is associated with a gradual decrease in WCC, that can be potentially detected by routine WCC monitoring. Hence, Japanese authors [7] state: *“We studied cases of agranulocytosis and granulocytopenia over the past 5 years at Kuma Hospital, where about 1500 new Graves’ patients start antithyroid drugs treatment annually, and found that nearly half of the patients were diagnosed with asymptomatic conditions by routine monitoring of granulocyte counts.”*

On the other hand, British and Australian endocrinologists [8, 9], entirely disagree with such strategy, and recommend immediate checks of WCC only in cases of pyrexia, severe sore throat or an overt infection. Furthermore, some authors directly criticize routine WCC monitoring, and state [8] that: *“Agranulocytosis can occur precipitously and without warning so routine white counts may engender a false sense of security.”* The British authors recommend a written information to be given to each patient receiving antithyroid drugs (together with a standard printed letter sent to General Practitioner or other health professionals), and strongly recommend that WCC checks should be done in primary care, rather than in specialist clinics. An example of such an information card is provided below (Fig. 1, from reference nr 8). Given my own personal experience, as well as difficulties to follow Japanese recommendations in Polish settings (for instance methimazole package insert in Japan includes a strong warning to check blood counts every 2 weeks for the first 2 months of therapy), I have adopted the British strategy in my personal medical practice.

**Declarations**

**Ethics approval and consent to participate**

Not applicable

**Consent for publication**

Not applicable

**Availability of data and materials**

Not applicable

**Competing interests**

The author declares no conflict of interests.

**Funding**

Not applicable

**Peer review**

This short paper underwent the journal’s standard peer review process for supplements.

**References**

1. Heidari R, Niknahad H, Jamshidzadeh A, Abdoli N. Factors affecting drug-induced liver injury: antithyroid drugs as instances. Clin Mol Hepatol. 2014;20:237-248.

2. Rivkees SA, Mattison DR. Ending propylthiouracil-induced liver failure in children. N Engl J Med. 2009;360:1574-1575.

3. Noh JY, Yasuda S, Sato S, Matsumoto M, Kunii Y, Noguchi Y, Mukasa K, Ito K, Sugiyama O, Kobayashi H, Nihojima S, Okazaki M, Yokoyama S. Clinical characteristics of myeloperoxidase antineutrophil cytoplasmic antibody-associated vasculitis caused by antithyroid drugs. J Clin Endocrinol Metab. 2009;94:2806–2811.

4. Korelitz JJ, McNally DL, Masters MN, Li SX, Xu Y, Rivkees SA. Prevalence of thyrotoxicosis, antithyroid medication use, and complications among pregnant women in the United States. Thyroid 2013; 23:758-765.

5. Andersen SL, Olsen J, Sen Wu Ch, Laurberg P. Birth defects after early pregnancy use of antithyroid drugs: A Danish nationwide study. J Clin Endocrinol Metab. 2013;98:4373-4381.

6. Napier C, Pearce SHS. Rethinking antithyroid drugs in pregnancy. Clin Endocrinol (Oxf). 2015; 82: 475-77.

7. Nakamura H, Miyauchi A, Miyawaki N, Imagawa J. Analysis of 754 cases of antithyroid drug-induced agranulocytosis over 30 years in Japan. J Clin Endocrinol Metab. 2013;98: 4776–4783.

8. Belchetz P, Hammond P. Mosby’s Color Atlas and Text of Diabetes and Endocrinology, Mosby Publishers, Edinburgh 2003, Chapter 17: Thyroid Disorders, pages 231-265.

9. Kennedy L , Basu A. Problems solving in endocrinology and metabolism. Oxford Clinical Publishers 2007. Chapter 42: Neutropaenia on Carbimazole, pages 210-214.

**Fig. 1 (abstract A10).**
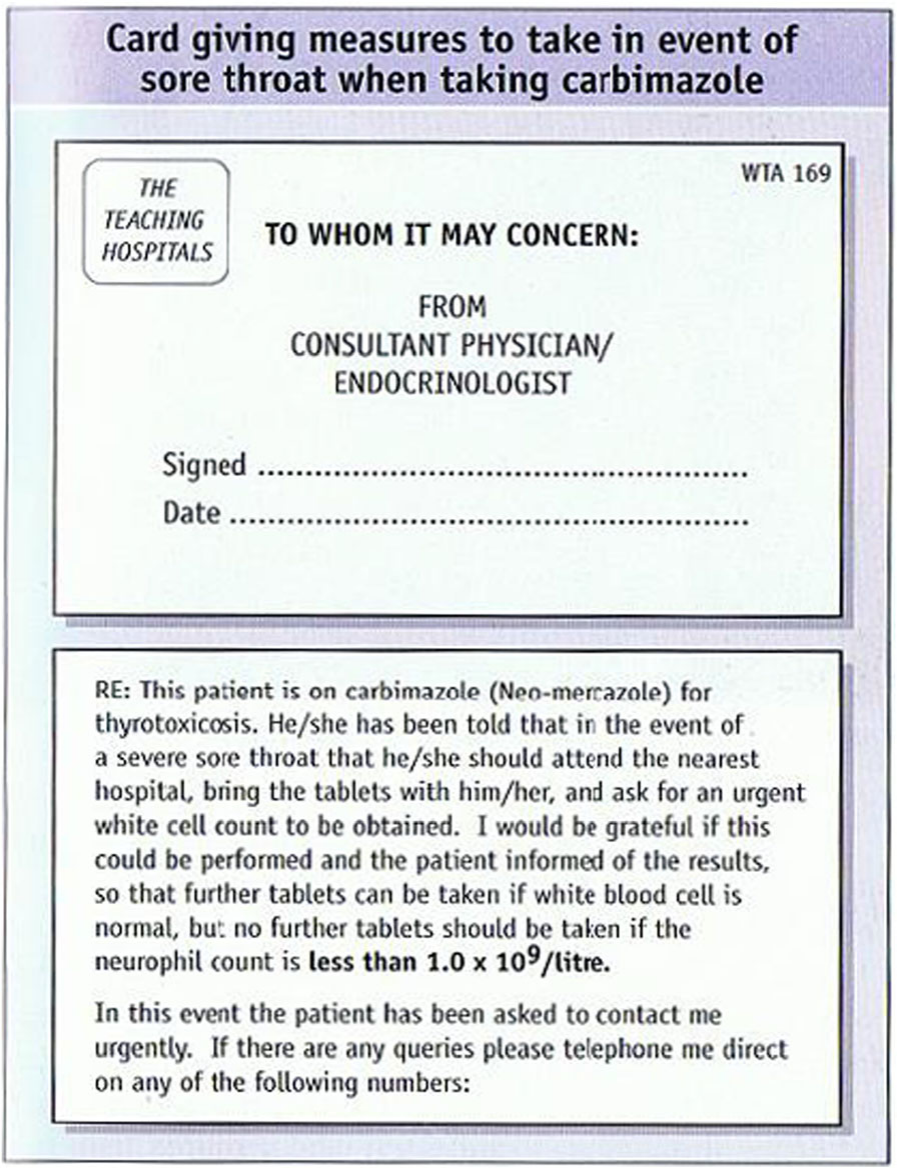
An example of information card on the risk of agranulocytosis (from Belchetz and Hammond, Mosby’s Colour Atlas and Text of Diabetes and Endocrinology, Mosby 2003, page 255)

### A11 Current modifications in preoperative ultrasound and cytological management of nodular goitre, thyroid cancer and thyroiditis

#### Andrzej Lewinski

##### Department of Endocrinology and Metabolic Diseases, Medical University of Lodz, Lodz, Poland

Currently, the most important clinical issue for a practicing endocrinologist is to answer the question whether the detection of thyroid nodule/nodules during physical examination or thyroid focal lesion/lesions in the ultrasound (US) scan provides the basis for referring the patient for surgery. The role of fine-needle aspiration biopsy (FNAB) in preoperative diagnostics of such cases is undeniable.

The fine-needle aspiration biopsy performance is recommended for each case of: 1) a palpable nodule corresponding to focal lesion with diameter of 5 mm or more, revealed during US examination, 2) an impalpable lesion with a diameter of 10 mm and more, visualised in US examination, 3) a lesion with suspicious US features, suggesting the malignancy (microcalcifications, “taller-than-wide” shape, increased irregular, chaotic central blood flows, hypoechogenicity, uneven thickness or absence of halo, solid composition (echostructure), the size greater than 3 cm in diameter, poorly defined and/or irregular margin, the rapid enlargement of lesion, as well as the presence of lymph nodes invasion.

Many years of personal experience allows us to say that the best way of conducting disease management is simultaneous careful consideration of both US findings and FNAB result, categorised according to The Bethesda System of Reporting Thyroid Cytology (TBSRTC) [1,2].

In addition to US data, one should account several disturbing clinical signs and symptoms, such as occurrence of hoarseness, dysphagia or pain which result from the presence of the thyroid tumour of firm consistency (Table 4). Also of importance is information on inherited diseases, with particular stress on multiple endocrine neoplasia 2A and 2B (MEN 2A and 2B), familial medullary thyroid carcinoma (FMTC) or familial non-MTC (FNMTC) in 1st degree relatives, as well as history of neck, head, chest or whole body irradiation - especially if it relates to the childhood. Another high risk factor of thyroid cancer that absolutely qualifies for further diagnostic investigation is patients’ age - under 20 and over 60 years. Furthermore, male sex is regarded as a risk factor for malignancy of thyroid lesions. The coexistence of essential “disturbing clinical signs and symptoms”, mentioned above, provide additional data indicating an increased risk of the malignant neoplastic disease.

As regards evaluation of ultrasound image pattern (US pattern risk), we have created the scoring system based on our own experience. For easier memorising the list of US features which should be assessed during US examination of thyroid nodules/focal lesions, we propose respective mnemonics [2,3].

Mnemonics are made up of the first letters of the names of these characteristics. The occurrence of calcifications (C) – especially microcalcifications, orientation (O) - “taller-than-wide”, when the antero-posterior diameter of a nodule is longer than its transverse diameter on a transverse or longitudinal plane, abnormal Doppler patterns (D), and echogenicity (E) - hypoechogenicity is awarded 1 point for each of these features. The absence of the regular "halo" (H), solid echostructure (E), the lesion largeness > of 3.0 cm (L), blurred margins (M) are associated with granting of 0.5 points for each of the features. Despite all the above characteristics, the attention should particularly be paid to the coexistence of augmentation (A) – the rapid enlargement of nodules and/or of focal lesions, accompanied by the presence of pathologically altered lymph nodes (L). The presence of these last two signs/symptoms out of ten (10) characteristics, is of crucial significance as it indicates a serious suspicion of the malignancy. Occurrence of each of these two features is associated with granting of 3 points. Our system, based on assigning the points to each US feature, is an attempt to evaluate risk stratification what – in turn – can help to select the optimal clinical decisions. The principle of assessment is to add points, which will allow classification of the lesions to particular groups of a different risk of malignancy (Table 5). However, in order to so happened, one should – as mentioned before – pay attention to the presence of other essential “disturbing clinical signs and symptoms” (Table 4).

It is to be stressed that introduction of the scoring system for “the disturbing symptoms and signs” is useless, because their assessment can be simply achieved by applying common sense. In other words, the occurrence of almost all of these symptoms by itself requires the execution of all possible diagnostic tests, followed by treatment implementation.

Having proposed our own point system, as a solution of the problem being - according to us - the most objective, I would like to recall how three other research groups coped with the problem in question.

Chilean authors, Horvath et al. [4], have developed a system of reporting ultrascan examination results of the thyroid nodules, stratifying risk of cancer during the clinical management (Thyroid Imaging Reporting And Data System – TIRADS), taking as an example the model of reporting data from US studies of the breast (BIRADS).

First, the authors [4] reviewed US findings of 362 thyroid nodules to define and specify their characteristics and establish US patterns. The following variables were considered: echostructure, echogenicity, shape, orientation, acoustic transmission, borders, surface, presence or absence of a capsule, calcifications, and vascularization.

Next, the authors prospectively correlated the FNAB results of another set of 500 nodules with the defined US patterns and generated a TIRADS group classification (TIRADS 1–6 for general thyroid pathology and TIRADS 2–6 for nodules).

The following categories were established: TIRADS1 – normal thyroid gland; TIRADS2 – benign conditions (0% malignancy); TIRADS3 – probably benign nodules (<5% malignancy); TIRADS4 – suspicious nodules (5–80% malignancy rate): 4a (malignancy 5-10%), 4b (malignancy 10-80%) was optional; TIRADS5 – probably malignant nodules (malignancy >80%); TIRADS6 – category included biopsy proven malignant nodules [4].

Park et al. [5] reported the results of their study in which they presented own system of data, reporting US features of the thyroid cancer.

The authors proposed an equation to predict the probability of malignancy in thyroid nodules based on 12 features of thyroid nodules, as noted on US.

This equation, and the stratification of its results into categories, should be useful in reporting the findings of US for thyroid nodules and in guiding management decisions.

Final regression analysis of the selected findings allowed the elaboration of a regression equation to generate logit of malignant thyroid nodule (z): z = -2.862 + 0.581X_1_ - 0.481X_2_ - 1.435X_3_ + 1.178X_4_ + 1.405X_5_ + 0.700X_6_ + 0.460X_7_ + 0.648X_8_ - 1.715X_9_ + 0.463X_10_ + 1.964X_11_ + 1.739X_12_.

The formula for the probability of malignancy of a thyroid nodule is as follows: P^us^ = 1/(1 + e^-z^), where: P^us^ - the probability of malignancy, as calculated by logistic regression using ultrasound features of the nodules; e - mathematical constant (e = 2.71828 . . .) [5].

Twelve independent variables were used in the equation in order to calculate the logit of malignant thyroid nodule: X_1_ - shape: taller = 1, wider = 0; X_2_ - perinodular halo: presence = 1, absence = 0; X_3_ - well circumscribed: presence = 1, absence = 0; X_4_ - microlobulation: presence = 1, absence = 0; X_5_ - infiltrative margin: presence = 1, absence = 0; X_6_ - marked hypoechoic: presence = 1, absence = 0; X_7_ - hypoechoic: presence = 1, absence = 0; X_8_ - homogeneous echotexture: presence = 1, absence = 0; X_9_ - mainly cystic: presence = 1, absence = 0; X_10_ - solid: presence = 1, absence = 0; X_11_ - microcalcification: existence = 1, absence = 0; and X_12_ - lymph node: abnormal = 1, normal = 0 [3].

Using this regression equation, P^us^ value of nodules classified as benign ones, was 0.07 to 0.23 with 95% CI, and 0.04 to 0.50 with 99% CI. P^us^ of nodules classified as malignant was 0.37 to 0.90 with 95% CI and 0.07 to 0.97 with 99% CI.

After calculating the P^us^ for each nodule, the authors stratified its distribution for each nodule and summarized the representative US findings to make the analysis applicable to a clinical setting: TUS 1 (P^us^ – 0-0.07) - inadequate cases, TUS 2 (P^us^ – 0.08-0.23) - benign, TUS 3 (P^us^ – 0.24-0.50) - indeterminate, TUS 4 (P^us^ – 0.51-0.90) – suspiciously malignant, TUS 5 (P^us^ – 0.91-1.0) – malignant, 99% CI [5].

The term “echotexture” requires explanation: it is defined as homogenous or mixed echogenicity, the latter due to the aggregation of multiple microcystic components intervening the solid component.

Korean authors divided US images of thyroid nodules into three categories: 1) nodules suspected of malignancy; 2) probably benign nodules; 3) indeterminate nodules [6].

The following features are characteristic for malignant processes: 1) “a taller-than-wide shape”; 2) a spiculated margin; 3) marked hypoechogenicity; 4) microcalcifications and macrocalcifications. The presence of at least one of these findings for malignancy defines a nodule as a suspicious malignant nodule [6].

The shape of a nodule (“taller-than-wide”, AP > TR diameter when imaged in the axial plane) has gained diagnostic importance for the differentiation of benign and malignant nodules since this was first described in a study by Kim et al. [7]. Also, in our studies, the shape was the strongest prognostic US feature of malignancy [1,2,3].

In contrast, a simple cyst, a predominantly cystic or cystic nodule with reverberating artifacts and a nodule with a spongiform appearance (especially with intervening isoechoic parenchyma) are defined as probably benign nodules [6].

Indeterminate nodules include nodules having US findings with neither malignant nor clearly benign characteristics. The US findings for an indeterminate nodule include: 1) isoechogenicity, hypoechogenicity, and hyperechogenicity, 2) an ovoid-to-round or irregular shape, 3) a smooth or ill-defined margin, 4) a rim calcification [4].

At the end of this presentation, I would like to quote a systematic review and meta-analysis of 31 studies from the years 1985 to 2012 (18 288 nodules), carried out by Brito et al. [8]. The results have led the authors to the following conclusions: 1) low - to moderate-quality evidence suggests that individual ultrasound features are not accurate predictors of thyroid cancer, 2) two features - cystic content and spongiform appearance, however, might predict benign nodules. However, this observation has very limited use in clinical practice, because of the rarity, especially of the latter characteristics.

Remonti et al. [9] - on the basis of a systematic review and meta-analysis of 52 studies retrieved by July 2012 (12 786 nodules) - concluded that there is no isolated US feature capable of predicting malignancy in thyroid nodules with acceptable diagnostic accuracy. However, the presence of some US features, such as: 1) a microcalcifications, 2) a taller than wide shape, 3) irregular margins, 4) central vascularization, 5) or absence of elasticity probably will identify nodules with an increased risk for malignancy.

In summary, the main goal of the preoperative diagnostics of thyroid tumours is the quickest and the most accurate selection of cases which should be surgically treated. Procedures proposed by us appear to be the simplest approach to the problem in question.

**Declarations**

**Ethics approval and consent to participate**

Not applicable

**Consent for publication**

Not applicable

**Availability of data and materials**

Not applicable

**Competing interests**

The author declares no conflict of interests

**Funding**

Not applicable

**Peer review**

This short paper underwent the journal’s standard peer review process for supplements.

**References**

1. Adamczewski Z, Lewinski A. Proposed algorithm for management of patients with thyroid nodules/focal lesions, based on ultrasound (US) and fine-needle aspiration biopsy (FNAB); our own experience. Thyroid Res. 2013;6:6.

2. Lewinski A, Adamczewski Z. Decision making for surgery in the suspect thyroid nodule (Proper application of ultrasound (US) and fine needle aspiration biopsy (FNAB) complement). Thyroid Intern. 2013;1:3-18.

3. Lewinski A, Adamczewski Z. Ultrasound and cytological diagnostics of thyroid - its proper application in case of coexisting disturbing clinical signs and symptoms, suggestive of active proliferative lesion. Thyroid Res*.* 2015;8 Suppl 1:A19.

4. Horvath E, Majlis S, Rossi R, Franco C, Niedmann JP, Castro A, et al. An ultrasonogram reporting system for thyroid nodules stratifying cancer risk for clinical management. J Clin Endocrinol Metab*.* 2009;90:1748-1751.

5. Park J-Y, Lee HJ, Jang HW, Kim HK, Yi JH, Lee W, et al. A Proposal for a thyroid imaging reporting and data system for ultrasound features of thyroid carcinoma. Thyroid 2009;19:1257-1264.

6. Moon W-J, Baek JH, Jung SL, Kim DW, Kim EK, Kim JY, et al. Ultrasonography and the ultrasound-based management of thyroid nodules: Consensus Statement and Recommendations. Korean J Radiol. 2011;12:1-14.

7. Kim EK, Park CS, Chung WY, Oh KK, Kim DI, Lee JT, et al. New sonographic criteria for recommending fine-needle aspiration biopsy of nonpalpable solid nodules of the thyroid. AJR Am J Roentgenol. 2002;178:687-691.

8. Brito JP, Gionfriddo MR, Nofal AA, Boehmer KR, Leppin AL, Reading C, et al. The accuracy of thyroid nodule ultrasound to predict thyroid cancer: systematic review and meta-analysis. J Clin Endocrinol Metab. 2014;99:1253-1263.

9. Remonti LR, Kramer CK, Leitão CB, Pinto LCF, Gross JL. Thyroid ultrasound features and risk of carcinoma: a systematic review and meta-analysis of observational studies. Thyroid 2015;25:538-550.

**Table 4 (abstract A11).** Two mnemonics for easier memorising the list of “disturbing signs and symptoms” that require inquisitive intense diagnostics, regardless of TBSRTC category and US pattern [2]

HARM		HASH	
H	Heredity	H	Hoarseness
A	Age	A	Ache
R	Radiation	S	Swallow
M	Male	H	Hardness

**Table 5 (abstract A11).** The scoring system of US features (patterns) assessed in thyroid nodules/focal lesions. Low risk US pattern – 0 < 3 points; intermediate risk US pattern – ≥ 3 < 7 points; high risk US pattern - ≥ 7 points [3]

CODE *(each feature – 1 point)*		
C	Calcifications	Max. no. of points – 4
O	Orientation	
D	Doppler	
E	Echogenicity	
HELM *(each feature – 0.5 point)*		
H	Halo	Max. no. of points – 2
E	Echostructure	
L	Largeness	
M	Margin	
AL *(each feature – 3 points)*		
A	Augmentation	Max. no. of points – 6
L	Lymph node involvement	

### A12 Serum selenium concentration in patients with autoimmune thyroiditis: own results and meta-analysis/review of literature

#### Anna Szeliga^1^, Adam Czyzyk^2^, Przemyslaw Niedzielski^3^, Miroslaw Mleczek^4^, Adam Maciejewski^5^, Anna Oczkowska^6^, Jolanta Dorszewska^6^, Katarzyna Lacka^5^

##### ^1^Student Scientific Society, Poznan University of Medical Sciences, Poznan, Poland; ^2^Department of Gynecological Endocrinology, Poznan University of Medical Sciences, Poznan, Poland; ^3^Department of Analytical Chemistry, Adam Mickiewicz University in Poznan, Poznan, Poland; ^4^Department of Chemistry, Poznan University of Life Sciences, Poznan, Poland; ^5^Department of Endocrinology, Metabolism and Internal Medicine, Poznan University of Medical Sciences, Poznan, Poland; ^6^Laboratory of Neurobiology, Department of Neurology, Poznan University of Medical Sciences, Poznan, Poland

###### **Correspondence:** Katarzyna Lacka

**Background**

Autoimmune thyroiditis (AIT) is a complex disease, which results from the interaction of both genetic predisposition and environmental triggers. Many different loci have been associated with increased susceptibility to AIT: both immunoregulatory (e.g. HLA class II, *CTLA-4*, *PTPN22*) and thyroid specific (*Tg*) genes variants. Among environmental factors some viral and bacterial infections, medications and inadequate micronutrients supply are believed to play a role. Selenium - a trace element built into the active centre of large protein family known as selenoproteins – is essential for protection against oxidative stress, for immune system balance maintenance and thyroid hormones synthesis and metabolism. Its deficiency is considered to be one of the factors underlying AIT etiopathogenesis.

**Aims**

The aim of our study was: 1) to assess the serum selenium concentration in patients with AIT and healthy volunteers in Polish population living in the Wielkopolska region, 2) to compare our results with data on selenium supply in other European populations.

**Materials and methods**

Forty seven euthyroid patients (41 women, 6 men) with AIT treated with L-thyroxine in dose of 1 μg/kg bw to 2 μg/kg bw was included in the study. Elevated TPO-Ab and/or Tg-Ab concentration and abnormalities typical for AIT found in the thyroid ultrasound were the criteria of AIT diagnosis, the coexistence of other diseases was applied as exclusion criteria. A control group consisted of 36 (24 women, 12 men) healthy, age and sex-matched volunteers. Patients with pathologies of the thyroid gland and other autoimmune, allergic, inflammatory or neoplastic diseases were excluded from the controls. The concentrations of TSH, TPO-Ab and Tg-Ab were determined by ELISA method. Serum selenium concentration in both groups was measured by electrothermal atomic absorption spectrometry (normal range 70-150 μg/L). The Mann-Whitney U test and Fisher exact test were used for statistical analysis (Statistica, StatSoft, Inc. 2011).

**Results**

The median serum Se concentration was 62.9 μg/L (ranging from 2.2 μg/L to 224.0 μg/L) in the patients group and 39.8 μg/L (ranging from 1.8 μg/L to 161.2 μg/L) in the control group (p = 0.029). A decreased selenium concentration was observed in 58% of the patients and in 72% of the controls (p = 0.29). There was no statistically significant difference (p > 0.05) in serum Se status in AIT group comparing to the results of other Polish, German, Austrian, Dutch and Greek populations with AIT, except from one Italian study, where significantly higher values were observed (p < 0.001)

**Conclusion**

In these preliminary results selenium deficiency is observed in the Polish population in both AIT patients and in the controls. Selenium supplementation should be considered in the deficiency state with further assessment of its results.

**Declarations**

**Authors' contributions**

Katarzyna Lacka: Concept of the study, collection of clinical material

Adam Czyzyk, Adam Maciejewski: Ultrasonographic evaluation of thyroid gland

Przemyslaw Niedzielski, Miroslaw Mleczek: Assesment of serum selenium concentration

Anna Oczkowska, Jolanta Dorszewska: Assesment of serum TSH, TPO-Ab, Tg-Ab

Anna Szeliga: Statistical analysis, elaboration of results

**Declarations**

**Ethics approval and consent to participate**

This study was approved by Local Ethics Committee, consent number 1035/13 Poznan University of Medical Sciences.

**Consent for publication**

Not applicable

**Availability of data and materials**

Available from the corresponding author on reasonable request

**Competing interests**

The authors declare no conflict of interests.

**Funding**

This study was funded by research grant number 502-05-02221355-50759 from University of Medical Sciences, Poznan, Poland

**Peer review**

This extended abstract underwent the journal’s standard peer review process for supplements.

**Fig. 2 (abstract A12).**
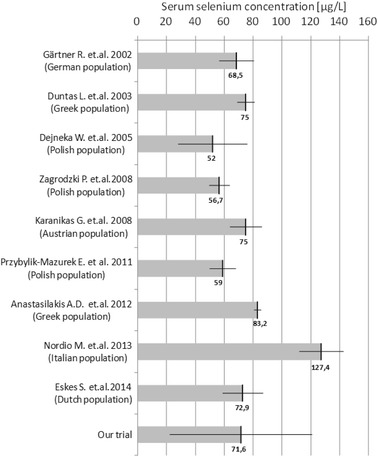
Comparison of serum selenium concentration between European populations with AIT and results obtained in our study

### A13 Osteoprotegerin in patients with thyroid disorders

#### Jerzy S. Tarach

##### Department of Endocrinology, Medical University, Lublin, Poland

**Background**

Osteoprotegerin (OPG) a member of the TNF receptor family, has been identified as a regulator of bone resorption because it acts as a decoy receptor by competing with the RANK ligand (RANKL *receptor activator of nuclear factor kappa B ligand*) for binding to the receptor activator of NF-κB (RANK) to exert an inhibitory effect on osteoclastogenesis, osteoclast differentiation and bone remodelling regulation. OPG is produced by a variety of tissues, including bone, the cardiovascular and immune system. Hitherto, it has been reported that the OPG/RANK/RANKL molecular triad plays an important role in several diseases, such as osteoporosis and cardiovascular diseases. OPG deficient mice (OPG-/-) develop severe osteoporosis and vascular calcifications. An increase in serum OPG is suggested to be a biomarker of the presence and severity of coronary heart disease and a risk factor for cardiovascular mortality. The physiological role of OPG, beyond the regulatory function of bone turnover, is the inhibition of cell apoptosis induced by inflammatory processes. OPG/RANK/RANKL are produced by variety of tissues including epithelial and mesenchymal cells. However, the role of RANKL/OPG in thyroid pathophysiology remains unclear. Anyway, it is known that hormonal disorders of the thyroid gland can be related to bone tissue disorders as well.

**Aims**

The aim was to identify the role of OPG in the context of the thyroid gland and thyroid disorders.

**Material and Methods**

PubMed database was searched for relevant articles from 2002 up to May 2015.

**Results**

It has been proven that OPG is produced in the thyroid gland by thyroid follicular cells and this synthesis may be modulated by thyroid autoimmunity. OPG mRNA levels are three times more abundant in surgical thyroid specimens of Graves’ disease compared with other thyroid disorders, OPG concentrations are higher in Graves' disease patients than in toxic nodular goitre ones and euthyroid subjects with Hashimoto's thyroiditis have increased levels of OPG compared to healthy controls. In the human thyroid follicular cell line XTC and in primary human thyroid follicular cells, OPG mRNA levels and protein secretion are up-regulated by TSH, IL-1β and TNFα. Neutralizing antibodies to OPG reverse the inhibitory effect of TSH on osteoclast differentiation evidencing that the TSH effect is at least in part mediated by elevated OPG. The association between thyroid function and OPG concentration has been shown. As well in hyperthyroidism as in subclinical and overt hypothyroidism, an increase in serum OPG levels has been reported which can be reversed / normalized by treating the diseases (anti-thyroid treatment or substitutive therapy). In thyrotoxicosis, an imbalance between bone resorption and formation is observed that results in bone loss and an increased risk for osteoporotic fractures. The low serum TSH, observed in thyroidectomized subjects on L-thyroxine therapy, is connected to an increase of bone turnover in postmenopausal women and men that is associated with a rise in OPG and a decrease of serum RANKL levels. Elevated concentrations of OPG in patients with hyperthyroidism are in relation to thyroid hormone excess and high bone turnover. The change of OPG levels is also associated with PTH concentration. Medical treatment of hyperthyroidism normalizes thyroid hormones and serum OPG levels in temporal relationship with the normalization of bone metabolism markers. Thus, it has been suggested that OPG may be involved in the cross-talking between thyroid function and bone metabolism in hyperthyroidism. Moreover, in a group of patients on suppressive hormone thyroid therapy with differentiated thyroid cancer, it has been proven that elevated levels of OPG (as well as changes in OPG/RANK/RANKL system), besides bone degradation, may influence on increased risk of cardiovascular diseases. It is also well known that hypothyroidism is associated with an increased risk for cardiovascular disease, impaired endothelial function and a rise in serum OPG is reported to connect to the severity of coronary heart disease and cardiovascular mortality. In hypothyroid patients, in whom elevated OPG concentrations are observed, during the course of the treatment, the absolute changes in OPG levels show a significant correlation not only with the changes in TSH but also with the changes in endothelium-dependent arterial dilation and plasma von Willebrand factor (a vascular injury marker). OPG levels are significantly higher in hypothyroid patients with silent myocardial ischaemia compared to hypothyroid subjects without vascular disease and in this group serum OPG is associated with flow-mediated endothelium-dependent arterial dilation. Even in euthyroid patients with Hashimoto's thyroiditis, serum OPG decreases dramatically after 6 months regular aerobic exercise training and is correlated with endotelium-dependent arterial dilatation. Therefore, it has been postulated that elevated serum OPG may be involved in the development of vascular dysfunction in hypothyroid patients. Concerning the role of OPG in carcinogenesis, it has been described that OPG/RANK/RANKL are expressed in the neoplasmatic thyroid gland - papillary and medullary carcinomas and macrovesicular adenomas by follicular cells and malignant parafollicular cells as well as in metastatic lymph node microenvironment. On thyroid cancer cells *in vitro*, OPG inhibits TRAIL-induced apoptosis and may promote tumour cell survival. Although OPG and TRAIL expression in benign and malignant tissues does not differ significantly, the expression of RANK protein appears to be suppressed and RANKL expression is increased in malignant thyroid tissue. Thus, this triad is hypothesized to play a role during pathogenesis of follicular and parafollicular tumours, as well as in the metastatic process of carcinoma cells to lymph nodes.

**Conclusions**

Undoubtedly, OPG plays a significant role in thyroid disorders. Therefore, further accurate assessment of the OPG relevance and its concentration in patients with thyroid diseases is needed.

**Declarations**

**Ethics approval and consent to participate**

Not applicable

**Consent for publication**

Not applicable

**Availability of data and materials**

Not applicable

**Competing interests**

The author declares no conflict of interests.

**Funding**

Not applicable

**Peer review**

This extended abstract underwent the journal’s standard peer review process for supplements.

## Other presentations: abstracts

### A14 MEN 1 syndrome, MEN 2 syndrome or neuroendocrine lung tumour with metastases to the thyroid and pituitary glands in 57-year-old patient treated in Department of Endocrinology in Szczecin

#### Elzbieta Andrysiak-Mamos, Elzbieta Sowinska-Przepiera, Ewa Zochowska, Bartosz Kiedrowicz, Agnieszka Kazmierczyk-Puchalska, Anhelli Syrenicz

##### Department of Endocrinology, Metabolic and Internal Diseases, Pomeranian Medical University, Szczecin, Poland

###### **Correspondence:** Anhelli Syrenicz

**Background**

In spite of current recommendations neuroendocrine tumours may cause diagnostic and therapeutic challenge. Frequently, patients find their way to the referral centres in the advanced stage of the disease.

**Case Report**

64-year-old male patient was sent to Department of Endocrinology in 2013 because of liver metastases of neuroendocrine tumour. He was operated in 2006 for the left lung tumour (typical *carcinoid*). For a few years he complained about flushing and diarrhoea. Since 2014 abdominal pain appeared and both abdominal ultrasound and CT showed multiple hepatic lesions described as metastases. Chest CT revealed neither local tumour recurrence nor right lung metastases. Histopathological examination of hepatic lesions confirmed neuroendocrine tumour with Ki67 from 17 to 31%. Somatostatin receptor scintigraphy reported increased expression of the receptors in liver and thoracic vertebrae defining NET metastases. High levels of chromogranin, 5-HIAA, calcitonin, CEA and AFP were detected. Thyroid ultrasonography showed a few lesions up to 10 mm in size. Medullary thyroid cancer was suspected on the basis of FNAB result and high serum calcitonin level and total thyroidectomy was performed. In histopathological examination there were multiple foci of neuroendocrine tumour with Ki67 from 7 to 22% and negative calcitonin immunoreactivity so the diagnosis of medullary thyroid cancer was ruled out. Subsequently histopathological diagnosis of a lung tumour from 2006 was verified as G2 (Ki67 – 12%), T3N1M0. The patient has been treated for 8 months with somatostatin analogues with good effect. Because of abnormal pituitary hormones levels, pituitary MRI was performed and showed a macroadenoma, which in histopathological analysis turned out to be a neuroendocrine tumour metastasis.

**Conclusions**

1. High serum levels of calcitonin and suspicion of medullary thyroid cancer in FNAB of thyroid lesions in patient with NET do not rule out thyroid metastases of NET. 2. Patients with NET need a follow-up with thyroid ultrasonography seeking for metastases.

**Consent for publication**

The authors acquired written informed consent from the patient.

### A15 Involvement of hMTH1 protein in thyroid carcinogenesis studied in thyroid cancer cell lines and tissue samples

#### Katarzyna D. Arczewska^1^, Joanna Drozdowska^1^, Wanda Krasuska^1^, Anna Stachurska^2^, Grazyna Hoser^3^, Miroslaw Kiedrowski^4^, Tomasz Stepien^5^, Hilde Nilsen^6^ and Barbara Czarnocka^1^

##### ^1^Department of Biochemistry and Molecular Biology, The Centre of Postgraduate Medical Education, Warsaw, Poland; ^2^Department of Immunohematology, The Centre of Postgraduate Medical Education, Warsaw, Poland; ^3^Department of Clinical Cytology, The Centre of Postgraduate Medical Education, Warsaw, Poland; ^4^Department of Pathology, Maria Sklodowska-Curie Memorial Cancer Centre and Institute of Oncology, Warsaw, Poland; ^5^Department of General and Endocrinological Surgery, Copernicus Voivodship Hospital, Lodz, Poland; ^6^Department of Clinical Molecular Biology, University of Oslo, Oslo, Norway

###### **Correspondence:** Katarzyna D. Arczewska

Oxidative stress promotes aging and contributes to multiple diseases, such as cancer and neurodegeneration. The thyroid tissue is an excellent model to study oxidative stress as active hydrogen peroxide (H_2_O_2_) production is required for thyroid hormone synthesis. Besides being a substrate in hormone synthesis H_2_O_2_ is a source of reactive oxygen species (ROS). Several studies revealed increased oxidative stress and oxidative DNA damage in thyroid tissue.

In presented research we were investigating the possible involvement of the hMTH1 protein, the major mammalian detoxifier of the oxidized DNA precursor, 8-oxo-dGTP, in the pathogenesis of differentiated thyroid cancer. Studies were performed on human thyroid tissues (cancerous and healthy), as well as normal (NTHY-ori 3-1), papillary (TPC1 and BCPAP) and follicular (FTC133) cancer thyroid cells using immunohistochemical, immunocytochemical, Q-RT-PCR, Western blot and flow cytometry techniques.

In agreement with earlier observations from other cancer types immunohistochemical analysis revealed hMTH1 protein overexpression in the thyroid tumours compared to normal thyroid tissues. Analysis of signalling pathways activated upon silencing of *hMTH1* gene expression revealed activation of MAPK signalling in NTHY cells. In addition, in NTHY cells increased Akt signalling was observed, whereas it was decreased in two of the cancer cell lines studied (FTC133 and TPC1). Although *hMTH1* gene knockdown had no influence on distribution of cell-cycle phases and apoptotic cell number up-regulation of p21 protein was observed in p53-proficient cell lines, NTHY and TPC1. Finally, phenotypic analysis revealed only minor influence of *hMTH1* gene silencing on proliferation rate and migration ability of NTHY and TPC1 cells.

Our data suggest that unexpectedly, differentiated thyroid cancer cells seem to tolerate hMTH1 down-regulation quite well, as evidenced by limited influence of hMTH1 silencing on their survival, proliferation, apoptosis and distribution of cell cycle phases, in spite of dependence of most of cancer cells types on hMTH1 protein expression.

Presented research was supported by Foundation for Polish Science PARENT/BRIDGE programme co-financed from European Union funds under the Innovative Economy Operational Programme 2007-2013.

### A16 Comparison of expression of selected tumour suppressor genes in thyroid cancer tissue (PTC, FTC, FA) and material from targeted fine needle aspiration biopsy

#### Karolina H. Czarnecka^1^, Michal Kusinski ^2^, Agnieszka Nadel^1^, Justyna Kiszalkiewicz^1^, Daria Domanska^1^, Monika Migdalska-Sek^1^, Dorota Pastuszak-Lewandoska^1^, Ewa Nawrot^1^, Krzysztof Kuzdak^2^, Ewa Brzezianska-Lasota^1^

##### ^1^Department of Molecular Bases of Medicine, I Chair of Internal Medicine, Medical University of Lodz, Lodz, Poland; ^2^Department of Endocrine, General and Vascular Surgery, Chair of Endocrinology, Medical University of Lodz, Lodz, Poland

###### **Correspondence:** Karolina H. Czarnecka

**Background**

Thyroid cancer is the most frequent endocrine neoplasia. Malignant transformation of nodular goitre (NG) can lead to the development of follicular adenoma (FA) that can progress into: papillary thyroid carcinoma (PTC) or thyroid follicular cancer (FTC). Ambiguous diagnosis as a result of preoperative differential diagnosis of thyroid nodules using fine needle aspiration biopsy (FNAB) is a significant clinical problem. It is important to search for new biomarkers differentiating follicular-cell derived thyroid tumours (FCDT), especially in case of FNAB with underdetermined cytology (Bethesda III-IV) or with „*neoplasma folliculare*” diagnosis.

**Aims**

Analysis of expression levels of selected tumour suppressor genes (TSG): *ARHI, CDKN2A, KCNQ1, CDH1* and *PTEN* in follicular tumour lesions FA, FTC and FVPTC (in tumour tissue examined postoperatively), and thyroid nodule biopsies taken during surgery. Assessment of usefulness of selected TSGs examination in patients with the "follicular neoplasm" in FNAB diagnosis.

**Materials and methods**

Biological material was obtained from 56 patients (aged 16 to 76 years) with preoperative diagnosis PTC/„*neoplasma folliculare*”. Histopathological classification: NG, n = 23; FA, n = 6; PTC, n = 22; FTC, n = 5. RNA was isolated from FNAB and cancer tissue (both collected during total thyroidectomy) using Universal RNA Purification Kit (Eurx, Poland). Qualitative and quantitative analysis of RNA was performed spectrophotometrically (BioPhotometer, Eppendorf). QPCR analysis was performed using Taq Man Low Density Arrays, TaqMan Array Micro Fluidic Card in 7900 HT Fast Real-Time PCR System (Applied Biosystems, USA). Relative gene expression (RQ) level of each TSG was assessed using ΔΔC_T_ method.

**Results**

Gene expression level of *CDKN2A, KCNQ1* and *CDH1* genes was at comparable level in tumour tissue and material from biopsies in the whole group, and groups of diagnosis (PTC, FTC, NG). Differences in expression levels of *ARHI* and *PTEN* genes were observed between cancer tissue and FNAB material, and in histopathological groups. *ARHI* and *PTEN* expression was significantly elevated in FNAB material when compared to cancer. There was no correlation of elevated gene expression with clinical or histopathological features.

**Conclusions**

Molecular analysis of the material obtained from intraoperative fine needle aspiration biopsy or *FNAB washes* (in case of sufficient volume of material) allows the assessment of the relative expression of several genes. Analysis of expression of *CDKN2A, KCNQ1* and *CDH1*, due to the comparable level of expression in tissues and biopsies, may have diagnostic significance and in the future may contribute to the improvement of differential thyroid lesion diagnosis carried out using FNAB.

### A17 Environment and the thyroid

#### Barbara Czarnocka

##### Department of Biochemistry and Molecular Biology, The Centre of Postgraduate Medical Education, Warsaw, Poland

Thyroid hormones (HT), T3 and T4 play a key role in the control of metabolism, through their contribution in the regulation of many crucial physiological processes in both children and adults. T3 and T4 are involved in the control of energy balance, growth and differentiation, brain development and function, circulation, osmotic and temperature control as well as in the regulation of other hormonal systems. The metabolism of the thyroid gland is precisely controlled by the active interaction between the hypothalamus-pituitary and thyroid gland maintaining a narrow range of normal T3 and T4 levels. Any disruption in the synthesis, secretion, transport and extrathyroidal T3/T4 metabolism can have an adverse effect. Disturbances in endocrine systems can be triggered by many factors such as diet, diseases or exposure to environmental chemicals and pollutants. 

In recent years, growing number of laboratory data, experiments on animal models, wildlife observations and human studies strongly suggests that many of the chemicals commonly found in the environment, have a negative effect on the thyroid gland and peripheral metabolism of thyroid hormones. Among thyroid disruptors (TDCs, *thyroid disrupting chemicals*) are industrial chemicals (polychlorinated biphenyls (PCBs), bisphenol A, flame retardants, phthalates, perchlorate and others), pesticides, and compounds used in the personal care products and cosmetics (among other UV-filters). TDCs can affect many steps of thyroid physiology and regulation from iodide uptake through thyroid hormones biosynthesis, transport to peripheral tissues, cellular uptake, peripheral deiodination, receptor activation, and finally their hepatic degradation and elimination. Hence, significantly more attention should be given to the identification of chemical compounds produced by humans, disrupting the function of the thyroid gland, which exert early and long-term effects on the adults and offspring and TDCs should be more widely noticed and recognized by the endocrinologists.

### A18 The efficacy of radioiodine therapy and a diagnosed thyroid disease – the own observations

#### Anna Maria Dabrowska^1^, Jolanta Kijek^2^, Jerzy S. Tarach^1^, Anna Torun-Jurkowska^3^, Beata Chrapko^2^

##### ^1^Chair and Department of Endocrinology, Medical University of Lublin, Lublin, Poland; ^2^Chair and Department of Nuclear Medicine, Medical University of Lublin, Lublin, Poland; ^3^Department of Mathematics and Medical Biostatistics, Medical University of Lublin, Lublin, Poland

###### **Correspondence:** Jerzy S. Tarach

**Background**

Radioiodine therapy is one of the radical and relatively safe methods of treatment of hyperthyroidism.

**Aims**

The aim of the study was to assess the efficacy of radioiodine therapy in patients with hyperthyroidism, depending on the diagnosed thyroid disease, termed as thyroid hormones concentrations, measured 12 months after radioiodine administration.

**Material and methods**

The analysis of hormonal and imaging findings, including isotopic results, in 1032 patients (865 F, 167 M) treated in the Department of Nuclear Medicine and in the Endocrinology Department, in Medical University of Lublin, during the eight-year period.

**Results**

In the studied group of patients, 484 subjects (85.33% of F) aged 55.42 ± 14.17 years had autonomous adenoma (AA), 366 subjects (80.60% of F) aged 46.68 ± 13.50 years - Graves' disease (GD), and in 182 participants (86.26% of F) aged 58.21 ± 12.96 years toxic nodular goitre (TNG) was diagnosed. In patients with AA: 80.79% achieved euthyroidism (81.60% of F with AA/76.05% of M with AA), 3.3% - hypothyroidism (3.39% of F with AA/2.82% of M with AA), and in 15.91% of patients (15.01% of F with AA/21.13% of M with AA) hyperthyroidism was observed; the efficacy of radioiodine therapy was estimated to be 84.10% (F – 84.99%; M – 78.87%). In patients with GD, euthyroidism was found in 24.32% (25.42% of F with GD/19.72% of M with GD), hypothyroidism - in 39.34% (39.32% of F with GD/39.44% of M with GD), and hyperthyroidism - in 36.34% of patients (35.26% of F with GD/40.84% of M with GD); the efficacy of radioiodine therapy was estimated to be 63.66% (F – 64.75%; M – 59.15%). Whereas, in subjects with TNG: 69.23% of patients achieved euthyroidism (66.88% of F with TNG/84.00% of M with TNG), 8.79% - hypothyroidism (8.92% of F with TNG/8.00% of M with TNG), and 21.98% had still hyperthyroidism (24.20% of F with TNG/8.00% of M with TNG); the efficacy of radioiodine therapy was estimated to be 78.02% (F – 75.80%; M – 92.00%).

**Conclusions**

Radioiodine therapy was the most effective in patients with AA and the least efficacy was achieved in GD subjects. Males responded better to treatment if the diagnosis of TNG had been made.

### A19 Graves' disease and Hashimoto's thyroiditis as components of autoimmune polyglandular syndromes

#### Anna Maria Dabrowska^1^, Jerzy S.Tarach^1^, Jolanta Kijek^2^

##### ^1^Chair and Department of Endocrinology, Medical University of Lublin, Lublin, Poland; ^2^Chair and Department of Nuclear Medicine, Medical University of Lublin, Lublin, Poland

###### **Correspondence:** Jerzy S.Tarach

**Background**

Autoimmune polyglandular syndromes (APS type 1, 2 and 3) are a group of diseases characterized by the coexistence of autoimmunological disorders of endocrine glands, other organs and systems as well.

**Aims**

The aim of the study was to determine the prevalence of Graves' disease and Hashimoto's thyroiditis as components of APS and their coexistence with other autoimmunological disorders.

**Material and methods**

Retrospective analysis of clinical symptoms as well as hormonal and imaging findings in 89 patients (70 F, 19 M) aged 50.0 ± 14.8 years, treated in the Endocrinology Department, Medical University of Lublin, between 2003 and 2015.

**Results**

Autoimmunological thyroid disease (ATD) was observed in 84.27% of subjects with APS: 100% with APS1, 86.36% with APS2, 82.26% with APS3. Graves' disease was diagnosed in 25.84% of patients with APS (19 F, 4 M): 13.64% with APS2 and 32.26% with APS3. In APS2, in 1 subject - type 1 diabetes and asthma were also found. In APS 3, Graves' disease coexisted the most often with: type 1 diabetes (60%), *vitiligo* (15%), *alopecia areata* (10%), Addison-Biermer anaemia (10%), *miasthenia gravis* (5%) and autoimmune hepatitis (5%). Hashimoto's thyroiditis was diagnosed in 58.43% of patients with APS (45 F, 7 M): 100% with APS1, 72.73% with APS2, 50% with APS3. In APS1, type 1 diabetes, *vitiligo* and *alopecia areata* were found as well. In APS2, Hashimoto's thyroiditis coexisted the most often with: type 1 diabetes, sarcoidosis, asthma and *vitiligo*. In APS3, besides Hashimoto's thyroiditis, type 1 diabetes (77.42%), *vitiligo* (22.58%), Addison-Biermer anaemia (16.13%), psoriasis (12.9%), *alopecia areata* (6.45%) and celiac disease, *colitis ulcerosa*, sarcoidosis, rheumatoid arthritis and asthma (3.23%) were observed.

**Conclusions**

Autoimmunological thyroid diseases are common components of APS, however Graves' disease occurs half as often as Hashimoto's thyroiditis. The coexistence of ATD and type 1 diabetes, *vitiligo* and Addison-Biermer anaemia are the most prevalent.

### A20 Prevention of Graves’ orbitopathy activation after radioiodine therapy; oral or intravenous steroids?

#### Helena Jastrzebska^1^, Magdalena Kochman^1^, Ewa Szczepanska^1^, Joanna Zgliczynska-Widlak^1^, Agnieszka Samsel^2^, Wojciech Zgliczynski^1^

##### ^1^Endocrinology Department of Medical Centre of Postgraduate Education, Warsaw, Poland; ^2^Ophthalmology Department, Childrens Hospital, Warsaw, Poland

###### **Correspondence:** Helena Jastrzebska

Radioiodine therapy may cause progression or development of Grave’s orbitopathy in 15-25% of patients. Moderate dose of glucocorticoids may protect orbital activation, however the optimal dose and duration of preventive steroids therapy is not established.

The aim of the study was to compare the effectiveness and safety of oral and intravenous glucocorticoids.

Eighty seven radioiodine treated Graves’ patients with mild orbitopathy, clinical activity score < 3/7, 72% (63/87) of them smokers, received prophylactic steroids, started at radiotherapy day. Sixty patients received oral prednisone, starting dose 30 mg or 20 mg daily for four weeks and then gradually reduced within 10 weeks, total dose 1200 mg and 790 mg respectively, and 27 patients received intravenous methylprednisolone 250 mg weekly for four weeks, total dose 1000 mg. Patients were assessed before and during 1-24 month after radioiodine therapy.

Graves’ orbitopathy progression evaluated using the clinical activity score was the primary outcome, side effects of steroid were the secondary outcome.

Graves’ orbitopathy progression and the indication for massive systemic corticotherapy was observed in both groups, in 8.3% (5/60) prednisone and in 7.4% (2/27) methylprednisolone patients. Activation occurred at smokers only independently of another risk factors such elevated serum T3 and TRAb levels or recurrent radioiodine therapy. The inconstant side effects including increase in body weight, elevated blood pressure, insomnia, and acne were noticed in prednisone group only. One diabetic patient from methylprednisolone group needed a marked increase of daily insulin dose.

Prophylactic oral or intravenous glucocorticoids are equally effective in prevention of Graves’ orbitopathy activation after radioiodine therapy but the intravenous method is safer.

### A21 Gluten-free diet and Hashimoto's thyroiditis

#### Roman Junik

##### Chair and Division of Endocrinology and Diabetology, L. Rydygier Collegium Medicum in Bydgoszcz, Bydgoszcz, Poland; Nicolaus Copernicus University in Torun, Torun, Poland

Euthyroid patients but with a high titre of autoantibodies, which consequently endocrinologist refused treatment, they feel abandoned by the official medicine. They start to walk to different paramedics, apply a variety of diets, including the paleo-diet or just gluten-free. Autoimmune thyroid diseases co-exist (not just of Hashimoto's thyroiditis but Graves' disease as well) and celiac disease stems from a common genetic predisposition (e.g. HLA-DQ2 and DQ8 haplotypes and other, e.g. CTLA-4). Autoimmune diseases of the thyroid gland and celiac disease is relatively rare: 4.8% (Collin), 3.3% (Sategna-Guidetti), 5.4% (Cuocco), 3.4% (Berti), 4.4% (Meloni), etc. Generally, it is considered that it is 2-5% of cases.

In the opposite direction it works a little differently, because in studied patients with celiac disease, frequency of accompanying autoimmune thyroid diseases was a bit higher: 6% (Reunala and Collin), 5.4% (Collin), and 14% (Counsell). Anti-TPO autoantibodies have been found in 29.7% (Veluzzi) and all types of antithyroid autoantibodies together were present in 41% (Kowalska, but this was children’s population). Perhaps this relatively high percentage of antithyroid auto-antibodies in patients with celiac disease is confused with the inverse situation, that is the presence of celiac disease (2-5%) in patients with autoimmune thyroiditis. Another problem is the fact that symptomatic celiac disease in adults is rare, the disease is insidious. Celiac disease can be resolved by performing a titre of IgA class antibodies: antiendomysial (EmA) and anti-tissue transglutaminase (anti-tTG).

You can also measure other antibodies. The gold standard is a biopsy of the mucous membrane of the proximal segment of the small intestine (Grzymislawski). It was confirmed that a gluten-free diet in patients with celiac disease with particular inflammation of Hashimoto could cause the disappearance of antithyroid autoantibodies (Ventura), but Mainardi has not found such a relationship. Sategna-Guideti concluded that a gluten-free diet causes relief of subclinical hypothyroidism in patients with celiac disease, while Cooper or Viljamaa such compounds are not observed. Patients with celiac disease and hypothyroidism under the influence of a gluten-free diet reduced the severity of primary hypothyroidism, and took the lower dose of thyroxine. It was found that a gluten-free diet is likely to be made as soon as possible, in order to reduce the risk of autoimmune thyroid inflammation join.

Summing up: the problem of a gluten-free diet affects patients with celiac disease, this diet is required. In the case of patients with autoimmune thyroiditis a gluten-free diet should take 2-5% of people, because so many suffer from celiac disease. If it is so make sense to force the remaining 95-98% of patients with Hashimoto's thyroiditis without celiac disease, a gluten-free diet for? Of course, in the case of suspicion of the presence of celiac disease follow a gluten-free diet and screening apply only in the case of the diagnosis of celiac disease.

### A22 The response of endocrine system to the critical illness: the thyroid gland

#### Dariusz Kajdaniuk^1,2,3^

##### ^1^Department of Pathophysiology and Endocrinology, Medical University of Silesia in Katowice, Katowice, Poland; ^2^Department of Endocrinology and Diabetology, Voivodship Specialistic Hospital No. 3 in Rybnik, Rybnik, Poland; ^3^Department of Nuclear Medicine and Endocrine Oncology, Center of Oncology - Maria Sklodowska-Curie Memorial Institute, Branch in Gliwice, Gliwice, Poland

Critical illness is defined as any life-threatening conditions requiring maintaining vital organ functions in order to avoid the upcoming death. Without modern intensive medical care the survival rate of critically ill patients is impossible. Critical illness is an extreme (the final) form of strenuous exercise. Biological reactions appearing in response to this condition are triggered immediately and on a larger, than in other clinical situations, scale. Stress response (critical illness) involves the reaction of the endocrine system (and also referred to as "the endocrine adaptation") and metabolic response. As a result of intensive medical care the survival rate of patients in states previously fatal has been increased, but currently more the patients "enter" in the chronic phase of critical illness, and requires a many weeks support/sustain the organ functions despite the removal of factors, which initiate the critical status. The lecture covers the data from recent years on the impact of serious non-thyroidal diseases on the function of the hypothalamic-pituitary-thyroid (HPT) axis and thyroid hormones metabolism that contributed to determining a pathomechanism of NTIS (non-thyroidal illness syndrome). The differences between acute and chronic phase of critical illness and between systemic inflammatory response syndrome (SIRS) (as sepsis) and conditions other than SIRS are discussed. Practical use: assessment the clinical status of critically ill patients, interpretation of laboratory tests, therapeutic treatment correction, endocrinological consultations in Intensive Care Unit.

### A23 Thyroid disorders and hypertension

#### Grzegorz Kaminski

##### Department of Endocrinology and Isotope Therapy, Military Institute of Medicine, Warsaw, Poland

There are three mechanisms in which thyroid hormones exert effect on the cardiovascular system:

1. Modulation of activity of nuclear receptors (triiodothyronine – T3).
2. Regulation of ion flow through the plasma membrane and within the cytoplasm (T3 and thyroxine – T4).
3. Altering responsiveness to sympathetic stimulation.

Hyperthyroidism results in increased heart rate (HR), left ventricular contractility and stroke volume (SV). This, in turn, according to Frank-Starling law (CI [cardiac index] = SV x HR), increases the CI value and greatly contributes to the development of hypertension. Thyrotoxicosis also produces vasodilatation which increases systolic pressure and decreases diastolic pressure. Blood pressure amplitude rise presents itself as *pulsus celer et altus* sign.

Cardiovascular symptoms of thyrotoxicosis can by relieved by beta-blocker (BB) therapy. In the course of BB treatment we observe heart rate and blood pressure normalization as well as alleviation of sympathetic overstimulation symptoms. However, as BB are not a homogenous group of drugs, their individual characteristics such as half life time or receptor and organ selectivity should be taken into consideration before the initiation of treatment. Their specific properties should also be considered – e.g. Nebivolol increases nitric oxide synthesis and reduces the oxidative stress which is of utmost importance in patients with diabetes and advanced atherosclerosis.

Hypothyroidism, on the other hand, results in CI and SV decrease. Increased vascular resistance and *pulsus parvus et tardus* sign, results of diastolic heart dysfunction, are as well observed. Impaired vasodilatation also serves as a main mechanism in diastolic blood pressure rise. Hence, the antihypertensive drugs used in hypothyroid patients should aim at dilating blood vessels and improving left ventricular diastolic function. Sino-atrial suppressors, as well as drugs promoting peripheral oedema are not advised. Angiotensin converting enzyme inhibitors (ACEI) and calcium-channel blockers seem to be the drugs of choice. However, similarly to BB, before treatment initiation their characteristics, as well as patient's comorbidities must be considered. Again, extra care should be given to the drug selection in patients with atherosclerosis. Only ACEI with well-documented favourable clinical outcome should be utilized in this group (e.g. long-acting Zofenopril containing sulphydryl group). Lecarnidipine, on the other hand, shows highest lipophilicity and lowest rate (<1%) of peripheral oedema among calcium-channel blockers which do not affect the pacemaker activity in the sino-atrial node.

### A24 Assessing the character of focal ^99m^Tc-MIBI-avid thyroid lesions discovered incidentally during myocardial perfusion scintigraphy

#### Grzegorz Kaminski, Krzysztof Giejda

##### Department of Endocrinology and Isotope Therapy, Military Institute of Medicine, Warsaw, Poland

###### **Correspondence:** Grzegorz Kaminski

**Background**

Thyroid diseases are the most common endocrine disorders. Although their detection rate is increasing, a considerable number of the above-mentioned conditions remain undetected. As population-wide screening tests would incur insupportable costs, there is a great need for highly sensitive and cost effective diagnostic tools. ^99m^Tc-MIBI is a radiopharmaceutical commonly used in the scintigraphic assessment of myocardial perfusion. It also accumulates in cancer cells, but may be as well used in the diagnostics of other, benign thyroid conditions.

**Aims**

The aim of the study was to assess the clinical significance of the focal ^99m^Tc-MIBI-avid thyroid lesions discovered incidentally during myocardial perfusion scintigraphy and to evaluate the utility of myocardial perfusion scintigraphy in the diagnosis of thyroid pathologies. We also aimed to develop an algorithm for the diagnosis of incidental thyroid lesions based on photon count number within defined regions of interest (ROI).

**Material and methods**

Focal ^99m^Tc-MIBI uptake in the thyroid was observed in 88/1041 patients referred to the Department of Nuclear Medicine of Military Institute of Medicine in Warsaw for ^99m^Tc-MIBI myocardial perfusion scintigraphy between 14.01.2009 and 31.10.2011. To include a patient in the study, ^99m^Tc-MIBI-avid thyroid lesion must have been reported by two nuclear medicine specialists. Quantitative analysis of thyroid scans included determining count number within ROI drawn over lesion as well as over two reference points: the contralateral thyroid lobe and lungs. All patients also underwent physical examination, thyroid ultrasound and laboratory tests measuring TSH, FT3, FT4, anti-TPO, TRAb, CEA, calcitonin, total calcium and phosphate levels. Based on the ultrasound results, selected patients were referred for fine-needle aspiration biopsy of thyroid lesions.

All patients participating in the study have signed a written consent form. The study has been reviewed and approved by the Bioethics Committee of the Military Institute of Medicine in Warsaw.

**Results**

Among 88 patients with focal ^99m^Tc-MIBI uptake thyroid disease was confirmed in 68 (77.2%) cases. Additional tests revealed: multinodular goitre (40 patients, 45.4%), solitary thyroid nodules (21 patients, 23.9%), 3 of which were autonomous, and autoimmune thyroid disorders (23 patients, 26.1%), among which were 7 cases of Graves' disease and 16 cases of Hashimoto's thyroiditis. Two patients (2.3%) were diagnosed with papillary thyroid carcinoma. ROI count number did not differ significantly between the group of patients with and without thyroid disease. In consequence, we were unable to develop a diagnostic algorithm based on photon count number within the ROIs. The gathered data also suggested that the quantitative evaluation of thyroid is not superior to the visual assessment.

**Conclusions**

1. Myocardial perfusion scintigraphy proved a valuable imaging method for detecting thyroid diseases. Thyroid pathologies diagnosed in patients with incidental focal 99mTc-MIBI uptake comprised thyroid nodules (74%), Hashimoto's disease (18%), Graves' disease (8%) and papillary thyroid cancer (2%) were the most common.
2. Quantitative and visual method of thyroid image interpretation did not yield significant differences in the accuracy of thyroid diseases diagnosis. In consequence, we were unable to create a diagnostic algorithm based on photon count number within ROI.
3. Thyroid assessment should be considered a standard procedure during stress myocardial perfusion scintigraphy as it shows usefulness in thyroid pathology diagnosis.

### A25 Hypothyroidism in women of childbearing age with respect to fertility; TSH ≥2.5 mIU/L in euthyroid women of childbearing age is associated with the increased oxidative damage to membrane lipids

#### Malgorzata Karbownik-Lewinska^1,2^, Magdalena Marcinkowska^1^, Jan Stepniak^2^, Andrzej Lewinski^1,3^

##### ^1^Polish Mother’s Memorial Hospital – Research Institute, Lodz, Poland; ^2^Department of Oncological Endocrinology, Medical University of Lodz, Lodz, Poland; ^3^Department of Endocrinology and Metabolic Diseases, Medical University of Lodz, Lodz, Poland

###### **Correspondence:** Malgorzata Karbownik-Lewinska

The increased level of thyroid antibodies is observed in at least 15% of women of childbearing age. In turn, the prevalence of hypothyroidism, especially of its subclinical form during preconception and gestational state, is estimated at the level of 10-15% or it is possibly higher. Hypothyroid patients on L-thyroxine replacement should be carefully monitored to keep TSH and thyroid hormones in recommended ranges before conception and during pregnancy. Untreated hypothyrodism has serious consequences, with decreased fertility being repeatedly documented. Whereas both overt and subclinical hypothyrodism constitute unquestionable indications for replacement therapy with L-thyroxine in most women of childbearing age, the cut-off values of thyroid tests still remain the point of debate. According to current recommendations, the estimated TSH upper limit of 2.5 mIU/L relates only to women planning pregnancy, pregnant and lactating women.

The aim of the study was to evaluate relationship between lipid peroxidation (LPO; index of oxidative damage to membrane lipids) and thyroid tests and other parameters, which may be affected by thyroid dysfunction, in euthyroid women of childbearing age.

Ninety nine (99) women, aged 18-48 years, without thyroid dysfunction (TSH 0.27–4.2 mIU/L, normal FT4 and FT3), were enrolled. Blood concentrations of malondialdehyde + 4-hydroxyalkenals (LPO index) were measured spectrophotometrically. Thyroid tests (TSH, FT4, FT3), thyroid antibodies and other laboratory parameters [cholesterol, HDL cholesterol (HDLC), LDL cholesterol, HDLC/cholesterol ratio, triglycerides, glucose, CRP, iron) were measured with standard methods.

Blood LPO level was higher in women with TSH ≥ 2.5 mIU/L than in women with TSH < 2.5 mIU/L. Positive correlation was fund between TSH concentration and LPO level. In the univariate regression analysis, blood LPO level did constitute the only factor associated with TSH ≥ 2.5 mIU/L. Abnormal HDLC/cholesterol ratio occurred more frequently in subjects with TSH ≥ 2.5 mIU/L. Additionally, positive correlation was found between LPO level and triglyceride concentration, whereas negative correlation was found between LPO level and HDLC concentration or HDL/cholesterol ratio.

In euthyroid women of childbearing age, TSH ≥ 2.5 mIU/L is associated with higher LPO and less favourable lipid profile, which suggests that TSH of less than 2.5 mIU/L should be maintain in the whole group of women of childbearing age.

### A26 Coexistence of well-differentiated thyroid cancer and thyroid lymphoma in patient after radioiodine treatment of toxic nodular goitre – case report

#### Monika Koziolek^1^, Anna Sieradzka^1^, Magdalena Lewandowska^2^, Maria Stepaniuk^3^, Bartosz Kiedrowicz^1^, Julita Duda^4^, Elzbieta Andrysiak-Mamos^1^, Anhelli Syrenicz^1^

##### ^1^Department of Endocrinology, Metabolic and Internal Diseases, Pomeranian Medical University, Szczecin, Poland; ^2^Department of Pathology, Pomeranian Medical University, Szczecin, Poland; ^3^West Pomeranian Oncology Center, Szczecin, Poland; ^4^Pomeranian Medical University Clinical Hospital No. 1, Szczecin, Poland

###### **Correspondence:** Anhelli Syrenicz

**Background**

Primary thyroid lymphoma is a rare thyroid neoplasm (about 1%), which develops often (70-80%) on the basis of Hashimoto’s thyroiditis. The most common thyroid cancer is the papillary cancer (>80%). Its follicular variant has nuclear morphological features and biological character of the papillary thyroid cancer but creates follicular structures.

**Case report**

68-year-old female patient, clinically and biochemically euthyroid, after I-131 radioiodine therapy for toxic nodular goitre was sent to the Endocrinology Outpatient Clinic with FDG PET/CT test result, performed as oncologic control after the ovarian cancer surgery performed in 2012. The patient had no complaints and there were no abnormalities in physical examination. Anti-thyroid autoantibodies titres were in normal range. FDG PET/CT revealed 9 mm hypodense lesion in the left lobe of the thyroid gland with SUV 5.62, which may correlate to adenoma, however malignancy could not be excluded. Thyroid ultrasonography showed in the right lobe a few, very small nodules and one bigger 10 x 9 x 7 mm solid hypoechoic lesion - with benign result of FNAB. It revealed also 17 x 12 x 8 mm diffuse hypoechoic area with increased flow in power Doppler on the border of isthmus and the left lobe and cytological and cytometric tests confirmed B-cell lymphoma. Oncology committee qualified the patient to surgery. Thyroidectomy was performed and histopathological examination revealed follicular variant of papillary thyroid cancer in 9 mm lesion in the right lobe and follicular lymphoma in 19 mm white infiltration on the border of isthmus and the left lobe. Currently, the patient receives R-CHOP chemotherapy.

**Discussion**

Microcarcinoma is very often an accidental finding in the thyroid gland removed because of other reasons than preoperatively confirmed cancer (25% or more).

Even though thyroid lymphoma belongs to rare tumours of thyroid gland, it needs to be kept in mind not only in patients with Hashimoto’s thyroiditis. In 80% of the cases in ultrasound images it presents as single hypoechoic lesion, sometimes almost anechoic, diffuse, with increased colour Doppler blood flow.

**Conclusion**

A patient after radioiodine treatment of toxic nodular goitre needs a regular endocrine follow-up including thyroid ultrasound and in case of finding new lesions FNAB is necessary.

**Consent for publication**

The authors acquired written informed consent from the patient.

### A27 The usefulness of *BRAF T199A* gene mutation and *RASSF1A* gene methylation detection in preoperative diagnosis of nodular goitre

#### Monika Koziolek^1^, Anna Sieradzka^1^, Ewa Wentland-Kotwicka^1^, Bartosz Kiedrowicz^1^, Milosz Parczewski^2^, Maria Stepaniuk^3,4^, Agnieszka Binczak-Kuleta^5^, Andrzej Ciechanowicz^5^, Elzbieta Andrysiak-Mamos^1^, Anhelli Syrenicz^1^

##### ^1^Department of Endocrinology, Metabolic and Internal Diseases, Pomeranian Medical University in Szczecin, Szczecin, Poland; ^2^Department of Infectious, Tropical Diseases and Immune Deficiency, Pomeranian Medical University in Szczecin, Szczecin, Poland; ^3^Department of Pathology, Pomeranian Medical University in Szczecin, Szczecin, Poland; ^4^West Pomeranian Oncology Center, Szczecin, Poland; ^5^Department of Clinical and Molecular Biochemistry, Pomeranian Medical University in Szczecin, Szczecin, Poland

###### **Correspondence:** Anhelli Syrenicz

**Background**

Molecular tests are used more commonly in qualifying a nodular goitre for the surgery, apart from the thyroid ultrasound and fine needle aspiration biopsy (FNAB), as on the basis of cytological examination conclusive diagnosis may not be always established. The research data indicate that in 10-15% of the FNAB not enough specimen is obtained to make a diagnosis and about 20% of biopsy analysis is in the range of cytological results, which demand postoperative histopathological verification.

**Aims**

Evaluate the incidence of *BRAF T1799A* mutation and *RASSF1A* promoter methylation level in cellular specimens derived from FNAB of thyroid nodules.

**Materials and methods**

The study population consisted of 80 women aged 18 to 65 years. Hormonal tests ruled out hyper- and hypothyroidism. Using autoantibodies and ultrasound autoimmune process was excluded. The study material was genomic DNA isolated from peripheral blood and thyroid bioptates. *BRAF* mutation was analyzed by standard methods of real-time amplification detection (real-time PCR) with the use of specific starters surrounding the mutated site. Pyrosequencing was used for the evaluation of *RASSF1A* methylation level.

**Results**

A significant positive correlation was demonstrated between mean methylation of four CpG islands of *RASSF1A* gene and thyroid tumour volume and its largest diameter (p < 0.05). *BRAF T1799A* mutation was found in 6/80 subjects (7.5%). In 5/6 mutation carriers benign nature of the thyroid nodules was confirmed by FNAB of the lesions, while non-diagnostic specimen was obtained from 1 subject but histopathological post-operative assessment diagnosed papillary thyroid cancer.

**Conclusion**

The results of genetic tests reported in our study indicate that the presence of *BRAF* mutation or higher *RASSF1A* methylation levels in FNAB cytology specimens of benign lesions may be useful in the assessment of oncological risk. Molecular tests may facilitate preoperative diagnosis of thyroid nodules and qualifying for surgery or conservative treatment.

### A28 Central hyperthyroidism in patients with thyrotropinomas - report of two cases

#### Maria Kurowska, Joanna Malicka, Piotr Denew, Agnieszka Zwolak, Monika Lenart-Lipinska, Jerzy S. Tarach

##### Chair and Department of Endocrinology, Medical University of Lublin, Lublin, Poland

###### **Correspondence:** Jerzy S. Tarach

**Background**

The first case of central hyperthyroidism due to thyrotropinoma [TSH-oma] was reported in 1960 and since then more than 450 cases of TSH-oma have been published. The prevalence of these tumours is about one case per million, and they account for about 0.5–3.0% of all pituitary adenomas. Most patients are diagnosed in the fifth-sixth decade of life with no gender difference. The majority of TSH-omas are large adenomas. Clinically they run with mild to moderately severe symptoms of hyperthyroidism. Patients are characterized by elevated levels of free thyroid hormones together with the lack of suppression of TSH secretion. Almost all TSH-omas express a variable number of somatostatin receptors what prompted the use of somatostatin analogues in their treatment. Somatostatin analogues induce a significant tumour mass shrinkage in about 40% of patients and normalization of circulating thyroid hormones in more than 90% of them. TSH-omas require differentiation with the resistance to thyroid hormone action syndrome (RTH).

**Aims**

To present two patients with central hyperthyroidism caused by TSH-secreting pituitary adenomas.

**Materials and methods**

The analysis of clinical picture and hormonal and imaging studies.

**Results**

**Case 1**. 69-year-old patient with pituitary macroadenoma [40 x 25 x 25 mm], incidentally detected in MRI performed during diagnosis of vertigo. All pituitary hormones were normal, but IGF-1 concentration was low [<0.25 ng/mL; n > 63.9]. Insufficiency of pituitary-gonadal axis was present [testosterone = 56.41 ng/dL; n > 242]. There were neither clinical signs of hyperthyroidism nor visual defects observed. The concentrations of thyroid hormones were: FT4 = 37.8 pmol/L [n: 12.0-22.0]; FT3 = 15.9 pmol/L [n: 3.0-7.0]; TSH = 1.61 mIU/L [n: 0.2-4.9]. Thyroid ultrasound revealed the presence of 73 ml nodular goitre. An increase of TSH of about 0.21 mIU/L during the TRH-TSH test excluded the diagnosis of RTH syndrome. The patient was treated with the use of thiamazol (periodically) together with long-acting somatostatin analogue (8 injections). The size of the tumour has not decreased. After reaching the normalization of thyroid function, the transsphenoidal surgery was conducted.

**Case 2** . 49-year-old patient, diagnosed due to weight loss, weakness and arrhythmias. He also didn’t complain of visual field defects. All pituitary hormones were normal. Failure of the pituitary-adrenal axis (ACTH = 23.17 pg/mL and cortisol circadian rhythm = 3.2----2.1 μg/dL) and pituitary-gonadal axis (testosterone = 95.8 ng/dL) were observed. Low IGF-1 level = 47.2 ng/mL (n > 94 ng/mL) was also certified. Thyroid hormones and TSH level were increased: FT4 = 5.91 ng/dL [n: 0.89-1.76]; FT3 > 20.0 pg/mL [n: 2.3-4.2]; TSH = 6.57 mIU/L [n: 0.55-4.78]. Thyroid ultrasound revealed the presence of nodular goitre. MRI picture showed 37 x 33 x 34 mm tumour mass localized in *sella turcica* and suprasellar region, penetrating to the pituitary stalk, optic chiasm, sphenoid sinus and right cavernous sinus. TRH-TSH test showed a decrease in TSH level. The patient has been initially treated with thiamazol followed by long-acting somatostatin analogue (4 doses). Partial regression of the tumour has been achieved. Currently neurosurgery is planned.

**Conclusions**

In parallel to the symptoms of central hyperthyroidism patients with TSH-omas also present other disturbances generated by the presence of macroadenomas. These signs can affect the course of the disease and require an acknowledgement in the diagnostic and treatment procedures. Patients with thyrotropinomas are characterized by different course of the disease and heterogenic response to therapy with somatostatin analogue.

**Consent for publication**

The authors acquired written informed consent from the patients.

### A29 Complexity of autoimmune thyroid disease (AITD) etiopathogenesis

#### Katarzyna Lacka

##### Department of Endocrinology, Metabolism and Internal Medicine, University of Medical Sciences, Poznan, Poland

Autoimmune thyroid disease (AITD) comprises autoimmune thyroiditis (Hashimoto’s thyroiditis, HT) and Graves’ disease (GD) including Graves’ ophthalmopathy (Graves’ orbitopathy, GO). In addition to these, we may also distinguish subclinical autoimmune thyroid disease characterized by the increased anti-thyroid antibodies without any clinical manifestations.

The frequency of AITD is estimated to be 5%, whereas the frequency of subclinical AITD is even higher, with a female: male ratio ranging from 5:1 to 10:1. This gender difference seems to be caused by parity (in relation to fetal microchimerisms), and the phenomenon of skewed XCI (skewed X- chromosome inactivation).

Although clinical manifestations of AITD are different; both are characterized by thyroid lymphocytic infiltration and the production of thyroid antibodies.

The etiopathogenesis of AITD is multifactorial and it is the result of an interaction between genetic susceptibility and environmental triggers leading to the breakdown of immunological tolerance and the development of AITD.

Among the environmental factors the most important are: smoking, iodine excess, selenium deficiency, bacterial (i.e. *Yersinia enterocolitica*) and viral (i.e. HCV) infections, various medications such as amiodarone, interferon alpha, alumtuzumab and others.

Susceptibility genes involved in the etiopathogenesis of AITD can be divided into thyroid-specific genes and immunoregulatory genes. Identified thyroid-specific genes are *TSHR and Tg.* The immune regulatory genes predisposing to AITD include: *HLA-DR class II, CTLA-4, P TPN22, CD25, CD40* and many others. It seems to be that HLA-DR beta –Arg 74 plays a critical mechanistic role in the development of AITD.

It is interesting that, with the exception of HLA-DR, most loci, which are associated with AITD, give low odd ratios (OR: 1.5-2.0). Thus, epigenetic modulations include DNA methylation, histone modifications and RNA interference by miR; may interact with genetic and environmental factors in the development of AITD.

### A30 Side effects of Y-90/Lu-177-DOTA-TATE therapy in the treatment of advanced medullary thyroid carcinoma

#### Dagny Lapinska, Kosma Wolinski, Magdalena Matysiak-Grzes, Aleksandra Klimowicz, Edyta Gurgul, Rafal Czepczynski, Maria Gryczynska, Marek Ruchala

##### Department of Endocrinology, Metabolism and Internal Medicine, Poznan University of Medical Sciences, Poznan, Poland

###### **Correspondence:** Marek Ruchala

**Background**

Medullary thyroid carcinoma (MTC) is often diagnosed in an advanced stage, with the presence of metastases. Among possible treatments the use of protein kinase inhibitors inspires hope, although actually their wider application is limited. Many surveys showed the expression of somatostatin receptors (SSTR) on the cells of the MCT, which justifies the fact of using somatostatin analogues, labelled with emitters of beta-radiation in therapy.

**Aims**

The aim of the survey was to assess the side effects of treatment with 90-yttrium (90-Y) and 177-lutetium (177-Lu) radiolabelled somatostatin analogue in patients with advanced MTC.

**Materials and methods**

The analyzed group includes 8 patients aged between 47 to 77 years with diagnosed advanced MCT, who were treated with four doses of 90Y/177Lu-DOTA-TATE, each of the activity 1.48/1.48 GBq, given at 12-week intervals. The toxicity of the treatment was assessed using CTCAE v. 4.03, considering biochemical parameters, such as haemoglobin level, neutrophils count, creatinine and liver transaminase levels. The parameters were assessed before treatment, after the treatment and after finishing the cycle.

**Results**

Among five patients who received the whole cycle of treatment, in three there was a decrease of 2 g/dL in haemoglobin levels observed, two of them had an additional increase of 0.2 mg/dL in creatinine levels, but none matched the criteria of an adverse event following CTCAE v. 4.03. Among the three remaining patients who still have not completed the cycle of treatment there were no adverse events observed between administration of the doses thus far.

**Conclusions**

In all patients the treatment was well tolerated, there were no adverse events matching the criteria of CTCAE. The lack of serious side effects may be caused by the interval between doses and the use of a protective infusion of amino acids before and after each treatment.

### A31 The role of cytokines TNF-α and osteoprotegerin in the diagnosis of a chronic autoimmune thyroiditis and Graves' disease in children

#### Hanna Mikos^1,2^, Marcin Mikos^3^, Barbara Rabska-Pietrzak^2^, Marek Niedziela^1,2^

##### ^1^Molecular Endocrinology Laboratory, Department of Pediatric Endocrinology and Rheumatology, 2nd Chair of Pediatrics, Poznan University of Medical Sciences, Poznan, Poland; ^2^Department of Pediatric Endocrinology and Rheumatology, 2nd Chair of Pediatrics, Poznan University of Medical Sciences, Poznan, Poland; ^3^Department of Pneumology, Allergology and Clinical Immunology, 3nd Chair of Pediatrics, Poznan University of Medical Sciences, Poznan, Poland

###### **Correspondence:** Marek Niedziela

**Background**

Chronic autoimmune thyroiditis (cAIT) and Graves' disease (GD) are the most common autoimmune disorders in children. TNF-α is associated with induction of inflammation and autoimmunity process. Osteoprotegerin is a soluble glycoprotein and a member of the tumour necrosis factor receptor (TNFR) family and plays an important role in bone homeostasis, autoimmune disorders and vascular diseases.

**Aims**

To determine concentrations of TNF-α and OPG in autoimmune thyroid disease (AITD) in children.

**Materials and methods**

We studied serum TNF-α and OPG (ELISA) in 22 newly diagnosed children with cAIT, 22 GD children and 20 healthy subjects with normal FT4, FT3, TSH and negative antithyroid Abs.

**Results**

In our study OPG concentrations were significantly higher in children with GD (median; IQR; range) (4.68 pmol/L; 2.86; 1.17-8.30) compared to control group (3.00 pmol/L; 1.98; 1.46-5.51)(p < 0.01). No significant difference was observed between TNF-α serum concentrations in cAIT (15.08 pg/mL; 21.94; 0.00-143.4) and GD group (13.63 pg/mL; 15.28; 0.00-28.5) vs control group (0.96 pg/mL; 12.81; 0.00-17.7) (p = 0.067). In children with hyperthyroidism we identified significant positive correlations between cytokines: TNF-α and OPG (r = 0.69; p < 0.01), while in the group with hypothyroidism, a significant correlation of TNF-α and ATPO (r = 0.54; p < 0.01). ROC curve analysis indicates that TNF-α differentiates children with hypothyroidism and healthy children AUC = 0.691; p = 0.034 with sensitivity of 54.5%, but a good specificity of 85%. On the contrary, OPG may be a marker of hyperthyroidism and exhibits a good discriminatory efficacy between group of hyperthyroid children and healthy children: AUC = 0.716; p = 0.017 at cut-off point of 4.54 pmol/L, with low sensitivity 54.5% but high specificity 95%.

**Conclusions**

Based on performed study we can consider TNF-α as a marker of hypothyroidism and OPG as a marker of hyperthyroidism in children with AITD.

### A32 Genetic background of multinodular goitre – *DICER1* syndrome

#### Marek Niedziela

##### Poznan University of Medical Sciences, 2^nd^ Chair of Paediatrics, Department of Paediatric Endocrinology and Rheumatology, Poznan, Poland

Nontoxic multinodular goitre (MNG) is frequently observed in the general population, but still little is known about the underlying genetic susceptibility to this disease. The terms adenomatous goitre, nontoxic nodular goitre and colloid nodular goitre are used interchangeably as descriptive terms when a MNG is found. Several hereditary tumour syndromes such as Carney complex, *PTEN* hamartoma tumour syndrome, Werner syndrome and *APC*-associated polyposis can be associated with non-neoplastic MNG however differentiated thyroid cancer can also occur. There are several factors that may be involved in the evolution of multinodular goitre: (1) primary - due to genetic abnormalities in several genes (the thyroglobulin-gene, the thyroid peroxidase-gene, the sodium-iodide-symporter-gene, the Pendred syndrome-gene, the TSH receptor-gene, the iodotyrosine deiodinase and the thyroid oxidase 2 gene3 leading subsequently to functional and structural abnormalities in growing goitres and (2) secondary such as elevated TSH as a potent stimulator of thyrocyte proliferation (induced by iodine deficiency, natural goitrogens, inborn errors of thyroid hormone synthesis), autoimmune thyroiditis (primary – intrathyroidal mechanism – disregulated apoptosis, and secondary - TSH-dependent nodules), smoking, stress, certain drugs, other thyroid-stimulating factors (IGF-1 and others) and endogenous factor (gender). Different somatic mutations are found in TSHR gene and the majority of mutations that are present in toxic adenomas are also found in toxic nodules in multinodular goitre. Germline mutations in the TSHR gene cause non-autoimmune autosomal dominant hyperthyroidism and in some patients with MNG.

Germline mutations in *DICER1,* a gene that codes for an RNase III endoribonuclease, disregulating miRNA expression pattern, have been identified in families affected by pleuropulmonary blastoma (PPB), some of whom include cases of MNG and gonadal tumours such as Sertoli-Leydig Cell Tumors (SLCTs). Moreover the other tumours such as cystic nephroma, embryonal rhabdomyosarcomas, Wilms tumors and other very rare entities, all comprise *DICER1* syndrome inherited in autosomal dominant pattern but with unknown penetrance. Cystic and hyperplastic thyroid abnormalities are a common finding in the *DICER1* syndrome with MNG, characterized by multinodular thyroid hyperplasia, being particularly prevalent in *DICER1* carriers, likely higher than that for neoplasms. Based on the literature it is hypothesized that this second somatic “hit” in *DICER1* is required in addition to a loss of-function germ-line *DICER1* mutation in order to initiate thyroid carcinoma development. A genetic counselling and testing should be offered to the family of the affected child.

### A33 Thyroid cancer in patients with acromegaly

#### Marek Ruchala, Ewelina Szczepanek-Parulska

##### Department of Endocrinology, Metabolism and Internal Medicine, Poznan University of Medical Sciences, Poznan, Poland

###### **Correspondence:** Marek Ruchala

Acromegaly is a chronic clinical condition resulting from prolonged exposure of the tissues to excess production of growth hormone by a pituitary tumour and, as a consequence, increased concentration of insulin-like growth factor (IGF-1). In the course of the disease characteristic changes of the patients’ appearance, as well as metabolic and systemic complications occur*.* One of the most important consequences of the disease is an increased risk of neoplastic transformation. Neoplasms, following cardiovascular and pulmonary diseases, constitute a third leading cause of death in patients suffering from acromegaly. Hence, proper diagnostics and introduction of adequate treatment of concomitant diseases are crucial in the management of patients with acromegaly, in order to improve their comfort of life and to avoid preterm death. The incidence of thyroid cancer and nodular goitre is high in acromegalic patients. Available data suggest that acromegaly is associated with a statistically significant increase in thyroid cancer risk compared to the general population. The risk is particularly high in subjects with an active or poorly controlled disease. Therefore, in order to detect neoplastic disease early and improve the prognosis, acromegalic patients require careful thyroid monitoring, including thyroid ultrasound examination and fine-needle aspiration biopsy, both at the diagnosis stage and during follow-ups. However, sonoelastography proves to be of limited value in the prediction of thyroid nodules malignancy diagnosed in acromegalic patients.

### A34 Role of homeobox transcription factor PROX1 in follicular thyroid cancer metastasis; correlation between PROX1 and AKT kinase signalling pathway

#### Magdalena Rudzinska, Joanna Ledwon , Kamila Karpinska, Maria Macios, Justyna Sikorska, Barbara Czarnocka

##### Department of Biochemistry and Molecular Biology, Center of Postgraduate Medical Education, Warsaw, Poland

###### **Correspondence:** Magdalena Rudzinska

The common hallmark of malignant tumours is their ability to metastasize. One of the key markers of lymphangiogenesis is **homeotic transcription factor-1 (Prox1)**. Several studies suggested that Prox1 protein plays an important role in carcinogenesis. The role of Prox1 has been described in several cancer.

The aim of our study was to determine Prox1 expression and examine its function in the biology of follicular thyroid cancer.

Our research was performed on cell lines derived from follicular thyroid cancer: FTC-133 (metastasis to the lymph nodes) and CGTH -W-2 (metastasis to sternum). Immortalized normal thyroid cell line (NTHY-ori-3) was used as a control. Prox1 mRNA and protein expression were determined using Q-RT-PCR, Western Blot and immunocytochemistry.

Role of Prox1 in the regulation of hallmarks of the malignant cell phenotype (migration, invasiveness, anchorage-independent growth and changes in cell morphology) was analyzed in cells upon Prox1 silencing or overexpression. To elucidate regulatory pathways involved in the observed phenotype we have applied Akt kinase inhibitor.

Prox1 transcript and protein levels were overexpressed in FTC133 and CGTH lines when compared with NTHY. Moreover, the protein was localized in the nucleus or both the nucleus and cytoplasm, respectively. Prox1 gene silencing decreased, whereas overexpression increased migration and the invasive potential. Changes in the Prox1 expression strongly influenced cytoskeleton and cells morphology. Moreover, we observed correlation between the Prox1 and the Akt kinase expression .

Our data suggest that the nuclear transcription factor Prox1 may play important role in the mechanism of follicular thyroid cancer cells metastasis.

Presented work was supported by grant 2012/07/B/NZ5/02444 and 213/11/N/NZ5/03394 from The National Science Center, Poland

### A35 Evaluation of TSH and FT4 levels in obese children

#### Malgorzata Ruminska, Ewelina Witkowska-Sedek, Beata Pyrzak

##### Department of Pediatrics and Endocrinology, Medical University of Warsaw, Warsaw, Poland

###### **Correspondence:** Malgorzata Ruminska

**Background**

Obesity in children leads to a number of metabolic and hormonal disorders. Among the hormonal disturbances the thyroid dysfunction is dominant. It is not clear whether it is the cause or the consequence of excess body weight.

**Aims**

To evaluate thyroid function in children with simple obesity compared to their peers with normal weight.

**Material and methods**

The study included 110 obese children (5-18 years) and 38 healthy peers. All patients underwent anthropometric measurements and laboratory tests (TSH, FT4, fasting glucose, triglycerides - TG, total cholesterol - TC, LDL-cholesterol, HDL-cholesterol). They calculated BMI-SDS, the waist to hip ratio (WHR) and the waist to height ratio (WHtR).

**Results**

Mean TSH concentrations in the obese children were statistically significantly higher than in children with normal weight (2.1 ± 1.0 μIU/mL vs. 1.5 ± 0.6 μIU/mL, p = 0.000). Mean concentrations of FT4 were comparable in both subgroups (1.0 ± 0.2 ng/dL vs. 1.0 ± 0.1 ng/dL, p = 0.589). In the whole study group TSH correlated with BMI-SDS (r = 0.333, p = 0.000), WHR (r = 0.326, p = 0.000), WHtR (r = 0.282, p = 0.001) and TC (r = 0.364, p = 0.000), LDL cholesterol (r = 0.333, p = 0.000), TG (r = 0.280, p = 0.001). FT4 concentration correlated only with body weight (r = - 0.213, p = 0.013) and the hip circumference (r = - 0.194, p = 0.024).

**Conclusions**

TSH concentrations observed in obese children were significantly higher compared with their peers with normal weight and correlated with the degree of abdominal obesity.

### A36 Quality of life in patients with Graves' orbitopathy

#### Nadia Sawicka-Gutaj

##### Department of Endocrinology, Metabolism and Internal Medicine, Poznan University of Medical Sciences, Poznan, Poland

Studies evaluating the quality of life (QoL) in patients with Graves' orbitopathy (GO) have shown that their QoL is profoundly impaired. Notable, QoL assessed by patient does not correlate with objective parameters such as duration of the disease, GO activity and severity. The prevalence of anxiety and depression is higher than in healthy population. GO has an impact on social and economic functioning, leading to temporary or permanent disability to work. GO should be considered a chronic disease because patients experience lower QoL even 11 years after therapy. The European Group on Graves' orbitopathy (EUGOGO) recommended using QoL questionnaire to assess patients' QoL as a part of clinical evaluation. In addition, EUGOGO recommended that the therapeutic decision in mild GO should be based on QoL measurement. The Polish version of GO-QoL questionnaire is now available and we recommend its use for evaluation of Polish GO patients.

### A37 Diagnostic difficulties in cytological assessment of thyroid nodules in patients with chronic autoimmune thyroiditis – case report

#### Anna Sieradzka^1^, Monika Koziołek^1^, Magdalena Lewandowska^2^, Ewa Wentland-Kotwicka^1^, Marcin Machaj^3^, Lilianna Osowicz-Korolonek^1^, Jakub Pobłocki^1^, Anhelli Syrenicz^1^

##### ^1^Department of Endocrinology, Metabolic and Internal Diseases, Pomeranian Medical University, Szczecin, Poland; ^2^Department of Pathology, Pomeranian Medical University, Szczecin, Poland; ^3^Department of Endocrinology and Internal Diseases, Autonomous Public Regional Joint Hospital in Szczecin, Szczecin, Poland

###### **Correspondence:** Anhelli Syrenicz

**Background**

Fine needle aspiration biopsy (FNAB) is a diagnostic method of choice in differential diagnosis of thyroid lesions, however it has some limitations. They may result from ambiguous cytological picture of the lesions connected with concomitant inflammatory process. The most common thyroiditis is Hashimoto’s disease. A common finding in thyroid follicular cells in this disorder is oxyphilic metaplasia. Nuclear features in such cells are mainly: large hyperchromatic nucleus, prominent nucleolus, sometimes vacuoles and intranuclear grooves, i.e. features of papillary thyroid cancer.

**Case report**

42-year-old female patient with asthma and rheumatoid arthritis, treated for a few years with levothyroxine because of chronic autoimmune thyroiditis, was sent to the Endocrinology Outpatient Clinic. She had no complaints and no abnormalities were found in the physical examination. Laboratory tests revealed TSH 2,10 uIU/ml (n 0,27-4,32), TPOAb 480 IU/ml (n 0-34). Thyroid ultrasound showed a solid, normoechoic lesion 12x8mm on the border of the right lobe and isthmus. FNAB was performed and the result was: suspicion of papillary thyroid cancer, characterized as Bethesda category V. The patient underwent thyroidectomy and eventually a benign lesion and chronic lymphocytic thyroiditis were reported in the histopathological examination.

**Discussion**

Cytological diagnosis of thyroid nodules, especially in patients with chronic autoimmune thyroiditis is sometimes a challenge for a pathologist and may be associated with formulating ambiguous or even false positive results. In the face of these limitations, it seems to be justified to include molecular procedures to the assessment of thyroid nodules in patients with chronic autoimmune thyroiditis.

**Conclusion**

Presence of the nodular chronic autoimmune thyroiditis hinders preoperative cytological diagnosis of thyroid lesions.

**Consent for publication**

The authors acquired written informed consent from the patient.

### A38 Influence of free triiodothyronine on the quality of life of patients with hypothyroidism treated with levothyroxine

#### Jerzy Sowinski, Nadia Sawicka-Gutaj, Paulina Ziolkowska, Marek Ruchala

##### Department of Endocrinology, Metabolism and Internal Medicine, Poznan University of Medical Sciences, Poznan, Poland

###### **Correspondence:** Jerzy Sowinski

In the clinical practice patients with hypothyroidism who are on levothyroxine (LT4) therapy often complain of symptoms characteristic for thyroid hormone deficiency despite having TSH level within the norm range.

The aim of the study was to assess the role of free triiodothyronine (FT3) concentration in the evaluation of the therapy of patients with primary hypothyroidism.

It was a prospective study. Patients included to the study group were on LT4 replacement therapy and had normal TSH level, and had complaints suggesting thyroid hormone deficiency.

The exclusion criteria were: thyroid cancer or another active neoplastic disease, pituitary gland insufficiency, liver and kidney diseases, use of medications: liothyronine, metformin, proton pump inhibitors, H2 antagonists, oral contraceptives, hormonal replacement therapy, calcium and iron preparations. In the study group the initial concentrations of TSH, free thyroxine (FT4), FT3 before and after increase of LT4 dose were measured. The quality of life initially and after increase of the dose was assessed using the questionnaire ThyPROpl.

Thirty three women of 37 patients completed the study (mean age 48.7 y). The initial mean concentration of TSH was 1.81 μU/mL ± 0.78 μU/mL, and FT4 16.73 pmol/L ± 1.85 pmol/L; median (IQR) FT3 3.94 pmol/L (3.74-4.29). After the increase of the dose of LT4 the mean ± SD: TSH - 0.64 μU/mL ± 0.49 μU/mL; FT4 – 19.43 pmol/L ± 1.55 pmol/L; FT3 – 4.75 pmol/L ± 0,54 pmol/L.

After the increase of LT4 dose the symptoms of hypothyroidism decreased, improvement of cognitive functions, emotional irritability, tiredness, anxiety, mood, daily activity, social activity, sexual functions, and goitre symptoms was observed. The lower FT3 concentration was associated with the anxiety, depression, emotional irritability, and goitre symptoms.

The increase of FT3 concentration in patients treated with LT4 is associated with the improvement of their quality of life.

### A39 Novel approach to etiopathogenesis of thyroid developmental abnormalities

#### Ewelina Szczepanek-Parulska, Bartlomiej Budny, Marek Ruchala

##### Department of Endocrinology, Metabolism and Internal Medicine, Poznan University of Medical Sciences, Poznan, Poland

###### **Correspondence:** Ewelina Szczepanek-Parulska

Thyroid embryogenesis is a complex and still unexplained process, with suspected of genetic background. Causative mutations were found in only a few percent of patients, mostly with severe thyroid developmental abnormalities leading to congenital hypothyroidism (agenesis, ectopy, hypoplasia).

In our study, we conducted sequencing and MLPA analysis of selected genes, known to be involved in thyroid embryogenesis. Moreover, an attempt to identify novel genetic factors through genomic studies (microarray, whole exome sequencing) was made. The studied group consisted of 34 patients with sporadic form of thyroid hemiagenesis (TH) and one family, where HT was transmitted from a mother to daughter. Several novel genes potentially involved in thyroid development were identified. They are engaged in the following biological processes: a) protein degradation via proteasome b) membrane transport c) cytoskeleton d) transcription regulation. Novel genetic alterations were found in the genes encoding different subunits of proteasome and also genes functionally related to proteosomal protein degradation. Haploinsufficiency of these genes may negatively impact developmental processes. Novel research indicate potential role of mutations in proteosomal subunits in numerous diseases (congenital, cardiovascular, neurological, autoimmune, neoplasms). The postulated mechanism of the disease indicates a toxic effect of undegraded protein accumulation. In conclusion, the identified alterations in patients with TH support the hypothesis of genetic background of the anomaly. Mutations in proteasomal genes might be causative in at least some portion of TH cases, and conceivably other thyroid developmental abnormalities. Our project is the first genomic study performed in such big cohort of patients with TH. Application of novel screening techniques allows for identification of novel candidate genes for TH and shed a new light on etiopathogenesis of thyroid dysgenesis, so far in majority reported to be associated with mutations in thyroid transcription factors.

### A40 Thyroid dysfunction and polycystic ovary syndrome

#### Malgorzata Trofimiuk-Muldner^1,2^

##### ^1^Department of Endocrinology, Jagiellonian University Medical College, Cracow, Poland; ^2^Endocrinology Department, University Hospital in Cracow, Cracow, Poland

Polycystic ovary syndrome (PCOS) and thyroid diseases are the most common endocrine dysfunction in women of reproductive age. Depending on criteria applied the frequency of PCOS is estimated at 8.7-17.8%. Hypothyroidism is diagnosed in about 3%, hyperthyroidism in 0.6% of menstruating women, however the thyroid antibodies positivity is encountered in up to 18% of that group. Many studies have confirmed that the thyroid autoimmunity is more frequent in PCOS women (OR of 4.8 for autoimmune thyroiditis in PCOS women according to Du et al. [1]), as well as higher prevalence of PCOS in women with autoimmune thyroiditis, regardless the TSH level. The nature of the association between thyroid dysfunction and PCOS has not been elucidated. The role of the genetic factors has been postulated. For example the polymorphism of fibrillin 3 (FBN3) gene, regulating the activity of the TGFβ cytokines has been suggested to be involved in the pathogenesis of both entities, however the true linkage has not been proven yet. The variants of GnRH receptor gene has been linked both with TSH level and insulin sensitivity, which pays an important role in PCOS pathogenesis. Polymorphisms of the genes encoding enzymes of steroid hormones metabolism pathway may also be involved. Also sex hormones themselves may at least partially explain the increased joint prevalence of thyroid autoimmunity and PCOS, which is characterized by relative hyperestrogenaemia. Estrogens, progesterone and prolactin are postulated to increase susceptibility to autoimmune disorders. Estrogen receptors are present in immune cells, such as Th1 and Th2 lymphocytes, and monocytes/macrophages. Increasing levels of estrogens are involved in shift to Th2-mediated immunity, increased regulatory T cell formation and increased activity of B cells. Arduc et al. [2] in a case-control study have shown positive correlation between TPO antibodies titre and both estrogen levels and estrogen/progesterone ratio.

It seems that autoimmune thyroiditis and subclinical hypothyroidism are not related to the PCOS fenotype. The other question is whether coexistence of autoimmune thyroid disease and/or hypothyroidism influences the PCOS-related metabolic risk. The currently available data are conflicting. Subclinical hypothyroidism has been shown to be related to reduced insulin sensitivity and increased LDL cholesterol and triglycerides levels in PCOS patients, however it is not clear if it is due to hypothyroidism itself or rather do to increased BMI and waist-to-hip ratio. However, for example Mueller et al. [3] have demonstrated that TSH level above 2 mIU/L can be an independent risk factor of insulin resistance.

The influence of PCOS treatment on hypothalamus-pituitary- thyroid axis should also be considered. Metformin is reported to lower TSH in hypothyroid patients, both under and without L-thyroxine (LT4) treatment. No such effect is seen in euthyroid patients not treated with LT4. Similar effects have been observed in PCOS patients. Thyroid diseases (particularly autoimmunity) may negatively influence PCOS treatment outcomes, particularly infertility. Ott et al. [4] for example have noticed higher titre of TPO antibodies in PCOS patients resistant to clomiphene citrate and metformin.

Concluding. PCOS and thyroid diseases, particularly thyroid autoimmunity, are frequent co-morbidities. The nature and consequences of this association still need to be explained. A PCOS patient, however, requires a close assessment of thyroid function to avoid possible negative effect of thyroid dysfunction on her general and reproductive health.

**References**

1. Du D, Li X. The relationship between thyroiditis and polycystic ovary syndrome: a meta-analysis. Int J Clin Exp Med. 2013;6:880-889.

2. Arduc A, Aycicek Dogan B, Bilmez S, Imga Nasiroglu N, Tuna MM, Isik S, Berker D, Guler S. High prevalence of Hashimoto's thyroiditis in patients with polycystic ovary syndrome: does the imbalance between estradiol and progesterone play a role? Endocr Res. 2015;40:204-10.

3. Mueller A, Schöfl C, Dittrich R, Cupisti S, Oppelt PG, Schild RL, Beckmann MW, Häberle L. Thyroid-stimulating hormone is associated with insulin resistance independently of body mass index and age in women with polycystic ovary syndrome. Hum Reprod. 2009;24:2924-2930.

4. Ott J, Aust S, Kurz C, Nouri K, Wirth S, Huber JC, Mayerhofer K. Elevated antithyroid peroxidase antibodies indicating Hashimoto's thyroiditis are associated with the treatment response in infertile women with polycystic ovary syndrome. Fertil Steril. 2010;94:2895-2897.

### A41 Mechanisms of sensitivity to thyroid hormones

#### Katarzyna Ziemnicka

##### Department of Endocrinology, Metabolism and Internal Diseases, Poznan University of Medical Sciences, Poznan, Poland

Thyroid hormones are essential regulators of the cell development and function. Their serum concentration is under precise control of hypothalamus-pituitary-axis via feedback regulatory system. Differences in the sensitivity to thyroid hormones might be responsible for some disturbances in psychosomatic development, low quality of life and not effective supplementation of thyroid hormones preparations.

Responsiveness to thyroid hormones is related to properly functioning system of hormones transmembrane transfer, deiodination and binding with specific nuclear receptor. Among the major groups of carriers contributing to thyroid hormones transport across the cell membrane one should mention: MCT (MCT8, MCT10), OATP (OATP1C, OATP1A2, OATP1A4) and LAT (LAT1, LAT2) and NCTP. Although MCT 8 has a specific affinity to thyroid hormones, the remaining proteins are transporting also aromatic amino acids (MCT10), organic anions (OATP), large, neutral amino acids (LAT). Very important elements of transport system are the escort proteins (e.g. 4F2hc) that drive synthesized proteins to the cell membrane.

The defect of gene coding thyroid hormone transporter, known as Allan-Herndon-Dudley syndrome, was reported. Clinical manifestation comprises intellectual disability, spastic paraplegia, involuntary movements, joint contractures and high serum T3, low serum T4 and normal or slightly elevated TSH. An inactivating recessive mutation of *SLC16A2* gene located on X-chromosome and coding for MCT8 transporter cause the syndrome. Polymorphisms in a gene coding OATP1C1 protein were also published and were referring to quality of life in patients treated with L-thyroxine.

Thyroid hormones that are effectively transported via cell membrane bind directly to specific nuclear receptors (T3) or undergo deiodination (T4 > T3) by deiodinase 1 or 2. Deiodinases activity might be modulated by genetic or environmental factors.

Until now, no crucial defects of deiodinases’ genes were reported, but it was found that autosomal recessive mutation in *SECISBP2* gene coding for protein contributing to incorporation of selenocysteine into selenoproteins in important way decrease deiodinase activity. Patients affected by this mutation present increased serum T4 concentration with increased TSH (although its level could be in the normal range) and decreased serum T3. Clinical manifestation comprises delayed growth, bone age and in some cases also impaired intellectual development, myopathy, hearing loss, infertility and immune deficiency. It has been demonstrated that polymorphisms of deiodinase 1 (*DIO1*) or deiodinase 2 (*DIO2*) genes might be related to variability of thyroid hormone concentration in serum, decreased quality of life in patients treated with L-thyroxine or to responsiveness to combined T3/T4 therapy.

Thyroid hormones bind to specific receptor in nucleus and mitochondria and mostly form heterodimer with retinoid X receptor. Thyroid hormones receptors are considered as transcription factors. The major isoforms showing high affinity to T3 are THRb1, THRb2 and THRa1. Binding of T3 to its receptor releases corepressors and leads to attachment of cofactors what triggers transcription. Apart from direct impact on transcription regulation, the thyroid hormones act via nongenomic (non-classical) pathway that is related to MAP kinase and Pi3K and regulate expression of FGF, GLUT1 or MCT4 proteins.

Resistance to thyroid hormones is a rare disorder with prevalence reaching approximately 1:40000 live births. This condition is caused by dominating mutations of *TRHb* gene, mostly leading to elevated T3, T4 and TSH being consequence of disturbances in negative feedback. Goitre and tachycardia are frequent symptoms. Abnormal cochlea development, impaired neural development and colour vision may also occur. Mutations within *TRHa* gene appears with lower frequency and lead to mild serum thyroid hormone disturbances. These defects are manifested by delayed bone development, chronic constipation, bradycardia and impaired neural development. In selected patients presenting similar signs and symptoms, which were previously described, no genetic defect was identified.

Increased sensitivity to thyroid hormones was described in mouse model lacking *THRa* gene. Recently, late onset resistance to thyroid hormones caused by various point mutations within *TRHb* gene was reported. It was manifested principally by goitre and palpitations, weakness or hyperactivity were not so common.

Except genetic factors, the sensitivity to thyroid hormones might be influenced by environmental factors and general patient’s health status. The responsiveness might be reduced in case of stress, injury, inflammation, low calorie diet, toxins or severe liver and kidneys diseases.

**References**

1. Refetoff S, De Wind LT, DeGroot LJ: Familial syndrome combining deaf-mutism, stippled epiphyses, goiter, and abnormally high PBI: possible target organ refractoriness to thyroid hormone. J Clin Endocrinol Metab 1967, 27:279-294.

2. Brent GA: Mechanisms of thyroid hormone action. J Clin Invest 2012,122:3035-3043.

3. Bernal J, Guadano-Ferraz A, Morte B: Thyroid hormone transporters – functions and clinical implications. Nat Rev Endocrinol 2015,11:406-417.

4. Moeller LC, Broecker-Preuss M: Transcriptional regulation by nonclassical action by thyroid hormone. Thyroid Res 2011,45 suppl. 1:56.

5. Fu J, Dumitrescu AM: Inherited defects in thyroid hormone cell-membrane transport and metabolism. Best Pract&Res Clin Endoclinol Metab 2014,28:189-201.

6. Macchia PE, Takeuchi Y, Kawai T, Cua K, Gauthier K, Chassande O, Seo H, Hayashi Y, Samarut J, Murata Y, Weiss RE, Refetoff S: Increased sensitivity to thyroid hormone in mice with complete deficiency of thyroid hormone receptor a. PNAS 2001,98:349-354.

7. Han R, Ye L, Jiang X, Zhou X, Billon C, Guan W, Gauthier K, Fang W, Wang W, Samarut J, Ning G: Endocrine 2015, doi:10.1007/s12020-015-0622-x.

### A42 Thyroid diseases and diabetes

#### Dorota Zozulinska-Ziolkiewicz

##### Department of Internal Medicine and Diabetology Poznan University of Medical Sciences, Poznan, Poland

Coexistence of thyroid diseases and diabetes is frequent and this has important clinical and therapeutic implications. Epidemiological data on the prevalence of thyroid diseases in the population with diabetes are divergent and indicate the percentage of between five and over thirty percent, similarly in type 1 and type 2 diabetes. As in the general population, the problem affects women more often than men.

Forty three percent of the participants Poznan Prospective Study (PoProStu) with type 1 diabetes have positive tyrosine peroxidase antibodies (TPO Ab), including significantly more often in women (53%) than men (21%). Epidemiological data indicate the advisability of screening for thyroid dysfunction in patients with diabetes.

The frequent co-occurrence of thyroid disease and diabetes is pathophysiological justified.

Autoimmune thyroid disease (AITD) and type 1 diabetes have a common genetic and environmental background. The association of major histocompatibility complex (MHC) and human leucocyte antigens (HLA) class II (HLA-DR) with these diseases has been known for over 30 years and remains the strongest, although finding a plurality of new consecutive genes associated with autoimmune process.

The negative impact of environmental factors, on the one hand interfere through epigenetic mechanisms, on the other metabolic phenomenon can be focused to insulin resistance, inflammation and oxidative stress. Both hyperthyroidism and hypothyroidism disrupt the regulation of energy homeostasis.

In people with diabetes, hyperthyroidism leads to faster absorption of carbohydrates from the gastrointestinal tract and promotes postprandial hyperglycaemia, increases hepatic glucose production, lipolysis and ketogenesis. Clinically, this translates into fluctuation of glycaemia, the occurrence of the dawn phenomenon, increased risk of ketoacidosis and increased demand for insulin.

On the other hand thyroid hormone deficiency is characterized by impaired glucose absorption from the gastrointestinal tract, reduced hepatic glucose production and decreased peripheral tissue glucose disposal. The clinical consequences in patients with diabetes are primarily associated with a decrease in insulin requirements, and increased risk of hypoglycaemia, including severe episodes.

Association of insulin resistance with thyroid diseases has not only functional but also structural background. It has been shown that insulin resistance has goitrogenic effect. In patients with abdominal obesity and metabolic syndrome larger volume of thyroid and more likely to have thyroid nodules was noticed. In addition, the association of insulin resistance with thyroid cancer is still discussed.

The significance of coexisting comorbidity of AITD and type 1 diabetes in assessment of the risk of development of late diabetic complications is uncertain. Surprisingly, it was demonstrated that adults with type 1 diabetes and positive thyroid antibodies develop less microangiopathy in comparison to group without AITD.

The higher knowledge in the theme of thyroid disease and diabetes, the more significant need for greater understanding and clinical sensitivity allowing for improvement of diagnostic and therapeutic effectiveness in the coexistence of these diseases.

